# Salt-Specific Gene Expression Reveals Elevated Auxin Levels in *Arabidopsis thaliana* Plants Grown Under Saline Conditions

**DOI:** 10.3389/fpls.2022.804716

**Published:** 2022-02-10

**Authors:** Lee Cackett, Carlo Vittorio Cannistraci, Stuart Meier, Paul Ferrandi, Aleš Pěnčík, Chris Gehring, Ondřej Novák, Robert A. Ingle, Lara Donaldson

**Affiliations:** ^1^Department of Plant Sciences, University of Cambridge, Cambridge, United Kingdom; ^2^Department of Molecular and Cell Biology, University of Cape Town, Rondebosch, South Africa; ^3^Center for Complex Network Intelligence, Tsinghua Laboratory of Brain and Intelligence, Department of Computer Science, Tsinghua University, Beijing, China; ^4^Center for Complex Network Intelligence, Tsinghua Laboratory of Brain and Intelligence, Department of Biomedical Engineering, Tsinghua University, Beijing, China; ^5^Center for Systems Biology Dresden (CSBD), Dresden, Germany; ^6^International Centre for Genetic Engineering and Biotechnology, Cape Town, South Africa; ^7^Laboratory of Growth Regulators, Institute of Experimental Botany of the Czech Academy of Sciences and Faculty of Science of Palacký University, Olomouc, Czechia; ^8^Department of Chemistry, Biology and Biotechnology, University of Perugia, Perugia, Italy

**Keywords:** salinity, salt stress, ionic, osmotic, auxin, IAA, plant, growth

## Abstract

Soil salinization is increasing globally, driving a reduction in crop yields that threatens food security. Salinity stress reduces plant growth by exerting two stresses on plants: rapid shoot ion-independent effects which are largely osmotic and delayed ionic effects that are specific to salinity stress. In this study we set out to delineate the osmotic from the ionic effects of salinity stress. *Arabidopsis thaliana* plants were germinated and grown for two weeks in media supplemented with 50, 75, 100, or 125 mM NaCl (that imposes both an ionic and osmotic stress) or iso-osmolar concentrations (100, 150, 200, or 250 mM) of sorbitol, that imposes only an osmotic stress. A subsequent transcriptional analysis was performed to identify sets of genes that are differentially expressed in plants grown in (1) NaCl or (2) sorbitol compared to controls. A comparison of the gene sets identified genes that are differentially expressed under both challenge conditions (osmotic genes) and genes that are only differentially expressed in plants grown on NaCl (ionic genes, hereafter referred to as salt-specific genes). A pathway analysis of the osmotic and salt-specific gene lists revealed that distinct biological processes are modulated during growth under the two conditions. The list of salt-specific genes was enriched in the gene ontology (GO) term “response to auxin.” Quantification of the predominant auxin, indole-3-acetic acid (IAA) and IAA biosynthetic intermediates revealed that IAA levels are elevated in a salt-specific manner through increased IAA biosynthesis. Furthermore, the expression of *NITRILASE 2* (*NIT2*), which hydrolyses indole-3-acetonitile (IAN) into IAA, increased in a salt-specific manner. Overexpression of *NIT2* resulted in increased IAA levels, improved Na:K ratios and enhanced survival and growth of Arabidopsis under saline conditions. Overall, our data suggest that auxin is involved in maintaining growth during the ionic stress imposed by saline conditions.

## Introduction

More than one billion hectares of land worldwide are considered saline, and this number is growing annually due to an increased occurrence of drought, the use of brackish water for irrigation and poor farming practices (FAO, IFAD, UNICEF, WFP, and WHO, 2018^1^; Ivushkin et al., 2019). Soils are considered saline when the electrical conductivity of a saturated soil extract (ECe) reaches 4 dS/m which is approximately equivalent to 40 mM NaCl. Salinity stress is a major factor limiting plant growth and performance, with moderate salinity between 4 and 8 dS/m reducing crop yields by 50-80% depending on the crop species (Zörb et al., 2019). It may not be possible to meet the demand for food from our rapidly growing population unless we develop salt tolerant crops. Despite substantial efforts, few salt tolerant transgenic crop plants have shown promise in field trials, likely due to the genetic complexity of salt tolerance (Morton et al., 2019), but also due to the inadequate identification of genes that promote plant growth and survival under saline conditions.

Salinity stress reduces crop yield by inhibiting shoot growth in two distinct phases (Negrão et al., 2017). Immediately following exposure to salt, the plant experiences rapid ion-independent, osmotic stress that dramatically reduces the shoot growth rate (Munns and Tester, 2008; Carillo et al., 2011). These effects are largely due to loss of turgor that prompts stomatal closure and, consequently, inhibits photosynthesis (Shabala and Cuin, 2007; Tilbrook and Roy, 2014; Julkowska and Testerink, 2015; Morton et al., 2019; Van Zelm et al., 2020). Such responses are also observed in plants exposed to abiotic stresses such as drought and cold, as well as in response to osmotic stress-inducing agents like sorbitol or mannitol (Ahmad et al., 2007). A second more gradual decline in plant growth is observed days (to weeks) after the initial salt exposure as a result of salt-specific ionic stress (Munns and Tester, 2008; Isayenkov and Maathuis, 2019). This corresponds with the toxic accumulation of Na^+^ and Cl^–^ ions in the aerial tissue of the plant (Assaha et al., 2017). Na^+^ is particularly harmful since it competes with the essential macronutrient, K^+^ for both uptake and binding sites on enzymes which leads to K^+^ starvation and inhibition of metabolic processes (Tilbrook and Roy, 2014; Assaha et al., 2017). Responses to the delayed ionic stress are largely salt-specific and include ion exclusion through active transport, ionic tissue tolerance and K^+^ retention (reviewed by Yang and Guo, 2018; Isayenkov and Maathuis, 2019). Although the osmotic and ionic stresses imposed by salinity are generally considered to be separated both spatially and temporally, it should be noted that more rapid salt-specific signal transduction and fast Na^+^-induced growth responses have been demonstrated in roots prior to Na^+^ having accumulated to toxic levels in the shoot (Choi et al., 2014; Van Zelm et al., 2020).

To date, few transcriptome studies have differentiated between the osmotic and ionic stresses imposed by salt (Shavrukov, 2013). Typically, plants have been treated suddenly with high concentrations of salt (150 mM NaCl and higher) and transcriptional responses measured after short time periods (24 h and less) (see for example Liu et al., 2013). The rapid exposure to high salt concentrations fails to mimic natural exposure to salinity stress and causes the plant to experience osmotic shock that, if severe enough, leads to cell plasmolysis (Munns, 2002; Shavrukov, 2013). Moreover, the short time periods used do not allow for Na^+^ ions to accumulate to toxic levels in the shoot such that the later ionic stress is induced. Therefore, it is likely that previous transcriptome experiments have identified genes involved in early osmotic shock/stress rather than genes specifically involved in plant growth and survival under saline conditions, i.e., plant salt tolerance (Abogadallah, 2010; Tang et al., 2011; Shavrukov, 2013; Goyal et al., 2016; Pavlović et al., 2018; Tani et al., 2018; Li et al., 2019).

Plant growth and adaptation to the environment is mediated by plant hormones (Gray, 2004; Bhatt et al., 2020). In particular, auxin or indole-3-acetic acid (IAA) influences almost every part of plant growth and development through its role in modulating cell expansion and elongation (Zhao, 2018). Auxin enhances plant growth by promoting cell elongation in shoot tissue, initiating lateral shoot and root formation, and mediating gravitropic responses. On the other hand, it inhibits the elongation of primary roots (Overvoorde et al., 2010; Alarcón et al., 2019). Therefore, controlling the location and concentration of auxin maxima is extremely important during plant growth and development and during adaptation to environmental stresses. Control of these maxima is achieved by fine-tuning IAA biosynthesis, IAA transport and IAA conjugation for storage and/or degradation (Korver et al., 2018), all of which are highly complex and regulated processes involving multiple genes. The biosynthesis of IAA is particularly complex and can occur *via* tryptophan-dependent (TD) and tryptophan-independent pathways. The TD pathway is the best understood and most widely accepted pathway for IAA biosynthesis, and has four proposed routes named for their key intermediates (IPyA, IAM, TAM and IAOx), (Ljung, 2013; Kasahara, 2016). The *YUCCA* (*YUC 1-11*) and *NITRILASE 1-3* (*NIT1-3*) gene families have been well characterized to convert IPyA and IAN into IAA, respectively (Normanly et al., 1997; Lehmann et al., 2017; Cao et al., 2019).

Recently, a series of elegant experiments have revealed a role for auxin in modulating root growth dynamics in response to salt. Under saline conditions, auxin modulates primary root (PR) elongation and lateral root (LR) initiation to adjust root growth and root system architecture (Ivanchenko et al., 2010; Overvoorde et al., 2010; Zolla et al., 2010; Koevoets et al., 2016; Fu et al., 2019). When presented with a salt gradient, the PR overcomes gravitropism to direct growth away from salt (Galvan-Ampudia et al., 2013). To achieve this, auxin accumulates and reduces cell elongation on the side of the PR that is exposed to the lower salt concentration. Cell elongation continues on the salt-exposed side of the PR root allowing the root to bend against gravity, away from high salt (Sun et al., 2008; Feng et al., 2016; van den Berg et al., 2016; Korver et al., 2018). Additionally, LR formation is inhibited by high levels of NaCl but stimulated by low levels of NaCl in an auxin-dependent manner (Wang et al., 2009; Zolla et al., 2010; Zhao et al., 2011; Ji et al., 2013; Liu et al., 2015). It has been proposed that this manipulation of the root system architecture enables plants to find more favorable growth environments, thus optimizing water and nutrient uptake and minimizing NaCl uptake (Duan et al., 2013; Julkowska et al., 2014; Koevoets et al., 2016). Despite extensive work on auxin modulation of root growth and architecture in response to salinity stress, we do not know how IAA influences the growth and development of the shoot under saline conditions.

In order to identify genes specifically involved in plant growth and survival under saline conditions, we compared the transcriptomes of *Arabidopsis thaliana* plants grown for two weeks on permissive concentrations of salt with plants grown on iso-osmolar concentrations of sorbitol. We identified osmotic and salt-specific genes and discovered a salt-specific role for auxin. Importantly, we measured the auxin metabolome and showed that IAA levels are elevated in plants grown under saline conditions compared to those grown under osmotic stress, again highlighting an ionic stress specific role for auxin. Finally, we showed that overexpression of the auxin biosynthetic gene, *NIT2*, resulted in improved survival and maintained growth, and a more favorable Na:K ratio in plants grown in saline conditions; which may be due to the increased IAA levels observed in the *NIT2* overexpression line.

## Materials and Methods

### Arabidopsis Seed Stocks

Seeds for the *NIT2* overexpression line (*35s:NIT2*) and its background (No-0) were a gift from Bonnie Bartel (Rice University, Houston, TX, United States) (Normanly et al., 1997). Seeds for the two T-DNA lines; *nit2-1* (SALK_201969C) and *nit2-2* (SAIL_681_H09) were obtained from the Nottingham Arabidopsis Stock Centre (NASC). Seeds for the *nit2-*RNAi line were a gift from Stephan Pollmann (Technical University of Madrid, Spain) (Lehmann et al., 2017).

### Arabidopsis Growth Conditions and Treatment

For all experiments, Arabidopsis plants were grown in a plant growth room under standard conditions (100 μM photons.m^–2^.s^–1^, 16-hr light/8-hr dark, 22°C, 50 to 60% relative humidity). Arabidopsis seeds were sterilized by shaking for 5 min in 70% (v/v) ethanol (EtOH). The EtOH was aspirated off before 10% (v/v) bleach, 0.02% (v/v) Triton X-100 was added, followed by another 10 min shaking. The seeds were washed five times in sterile dH_2_O and resuspended in 0.5% (w/v) phytagel. All plant growth was carried out in *Arabidopsis thaliana* salt (ATS) media which contains 5 mM KNO_3_, 2 mM MgSO_4_.7H_2_O, 2 mM Ca(NO_3_)_2_.4H_2_O, 50 μM FeNaEDTA and 1 × micronutrients (70 μM H_3_BO_3_, 14 μM MnSO_4_.H_2_O, 0.5 μM CuSO_4_.5H_2_O, 1 μM ZnSO_4_.6H_2_O, 0.2 μM Na_2_MoO_4_, 10 μM NaCl and 0.01 μM CoCl_2_.6H_2_O). After autoclaving, KPO_4_ buffer pH 5.7 was added to a final concentration of 2.5 mM (Haugh and Sommerville, 1986; Wilson et al., 1990). For Arabidopsis growth in petri dishes, sterilized seeds were sown onto ATS-agar (6% w/v) supplemented with 50, 75, 100, or 125 mM NaCl or iso-osmolar concentrations of sorbitol (100, 150, 200, or 250 mM sorbitol). Within each experiment, treatments or genotypes included four plates sown with 50 seeds. Seedlings were grown for two weeks, after which the number of surviving seedlings on each petri dish was counted (defined as a seedling which had developed true leaves) and the total mass of seedlings per plate measured. For hydroponic growth, Arabidopsis plants were grown using the Araponics system (Liége, Belgium) for 3 weeks in 1/4 strength ATS media and then transferred onto ATS media supplemented with 0 mM (control) or 75 mM NaCl for a further week. Shoot and root tissue from the four-week-old plants was subsequently harvested for downstream experiments.

### RNA Extraction, Quantification, and cDNA Synthesis

RNA was extracted from ± 100 mg of plant tissue (fresh weight) using the Trizol^®^ Reagent according to the manufacturer’s instructions (Invitrogen™, Carlsbad, CA, United States). Following RNA extraction, DNase treatment was performed using the RNase-Free DNase kit (Qiagen, Germany) and DNase treated RNA was cleaned up using the RNeasy^®^ Mini Kit (Qiagen, Germany). The RNA concentration and quality were then analyzed using the Nanodrop^®^ ND-1000 spectrophotometer (NanoDrop Technologies, Wilmington, CA, United States) and by visualization after denaturing gel electrophoresis. cDNA was synthesized from 1 μg of RNA using SuperScript™ III Reverse Transcriptase and oligo dT primers according to the manufacturer’s instructions (Invitrogen, Paisley, United Kingdom).

### RT-qPCR Gene Expression Analysis

The KAPA SYBR^®^ FAST qPCR Kit (Kapa Biosystems, Roche, Switzerland) was used according to the manufacturer’s guidelines (without 50X ROX High/Low). Experiments were performed on the Corbett Rotor-Gene 6000 HRM Real Time PCR machine (Qiagen, Netherlands). The calculated concentrations extrapolated from the RT-qPCR standard curve were normalized to the SAND reference gene (AT2G28390) (Hong et al., 2010). RT-qPCR primer sequences (5′- 3′):

*SAND:* F-CAGACAAGGCGATGGCGATA, R-GCTTTCTCT CAAGGGTTTCTGGGT,

*NIT2:* F-CTCCCGCCACTCTAGAAAAG, R–AATAGCAGA AGCATGGTACTTGC,

*SZE2*: F-TCCCTTCAAGTTCAGTGGAGC, R- TCTCATTG ATGCAGCCTTCGT,

*TIP2.3:* F- TAATGGCAAGAGCGTACCGAC, R- ACCAAT GCAAAGGTCACAACG,

*AMI1:* F- CAACTTCTACTTCCTCGTCGC, R- CTCCGTT TATACTGTAAGCCATTT.

### Salt-Specific Transcriptome Analysis

Col-0 was grown for two weeks in petri dishes on ATS-agar (6% w/v) supplemented with 50, 75, 100 or 125 mM NaCl or iso-osmolar concentrations of sorbitol (100, 150, 200, or, 250 mM sorbitol). Total RNA was extracted from whole seedlings and submitted to the genomics facility at the King Abdullah University of Science and Technology (KAUST). Microarray analysis was performed using the Arabidopsis (V4) Gene Expression Microarray, 4 × 44k microarray chip (Agilent Technologies, CA, United States). All protocols were followed according to the manufacturer’s guidelines in the Agilent One-Color Microarray-Based Gene Expression Analysis (Low Input Quick Amp Labeling) Protocol. The microarray data was quantile normalized for each sample followed by *z*-score transformation of each gene (to allow genes to be compared on the same scale). Logarithmic scaling of the absolute value of the data was performed to stabilize variance. The data was then separated into three groups of samples: untreated control (3 samples), all NaCl treatments (4 NaCl treatments with 3 samples each = 12 samples) and all sorbitol treatments (4 sorbitol treatments with 3 samples each = 12 samples). The Mann-Whitney *U* test (Mann and Whitney, 1947) was used to compare gene expression between groups. This generated a *p*-value for each gene in the dataset. The list of *p*-values was adjusted for multiple hypothesis testing by Benjamini and Hochberg correction (Benjamini and Hochberg, 1995), and only genes with a *p*-value < 0.01 were considered significantly different between the two groups. Two independent Mann-Whitney U tests were performed to generate two lists of genes: list A (untreated control vs. NaCl) and list B (untreated control vs. sorbitol). To generate the salt-specific gene list, the genes present in list A, but not list B were identified (A/B). To generate the osmotic gene list, the genes present on both list A and list B were identified (intersection of A and B). Heat maps were generated using TMev (Howe et al., 2010).

### Gene Ontology Enrichment Analysis

The topGO (Alexa et al., 2006) R Bioconductor package was used to test for enrichments in Gene Ontology (GO) terms associated with the differentially expressed genes. The GO graph structure was generated using both the “classic” and “weight.01,” algorithms and the Fisher exact test was used to identify enriched terms.

### LC-MS/MS Analysis of Indole-3-Acetic Acid and Indole-3-Acetic Acid Metabolites

Col-0 plants were grown for two weeks in petri dishes on ATS-agar (6% w/v) supplemented with 50, 75, 100, or 125 mM NaCl or iso-osmolar concentrations of sorbitol (100, 150, 200, or 250 mM sorbitol). No-0 and *35s*:*NIT2* plants were grown for two weeks in petri dishes on ATS-agar (6% w/v) supplemented with 0 or 100 mM NaCl. After two weeks of growth, 10-15 mg of tissue (whole seedlings) from each genotype and treatment was collected into pre-weighed 2 ml microcentrifuge tubes. Samples were immediately frozen in liquid nitrogen and then freeze dried (ThermoVac Industries Freeze dryer, FD-3 model, serial no. 1867) and IAA metabolites measured as previously reported (Novák et al., 2012). For each treatment five independent biological replicates were included in the analysis.

### Inductive Coupled Plasma – Optical Emission Spectrometry Analysis of Ion Content

No-0 and *35s*:*NIT2* plants were grown hydroponically in 1/4 strength ATS media for three weeks and then transferred onto ATS media supplemented with 0 mM (control) or 75 mM NaCl for a further week. For each treatment, four No-0 or four *35s*:*NIT2* plants were pooled, the roots rinsed three times with dH_2_O, and the root and shoot tissue harvested separately, then dried at 60°C. This set up was repeated six times to give a total of six biological replicates (each replicate being a pool of four plants). Dried shoot and root samples were submitted to the Analytical Laboratory, Department of Chemical Engineering (University of Cape Town, South Africa) where the samples were ashed, weighed into Teflon containers, and acid digested as follows: 4 ml 55% H_2_O HNO_3_ (v/v) was added to the samples which were then boiled to near dryness using a hot plate. This process was repeated until the samples were completely digested. The digested samples were diluted to 100 ml using dH_2_O. A Mars 5 Microwave digester (CEM Corporation, Charlotte, NC, United States) was used to heat the samples under pressure at 180°C for 30 min. Following digestion, samples were diluted in dH_2_O according to the initial mass of the sample. All samples were then filtered using a 0.2 μm filter. Sodium (Na^+^) and potassium (K^+^) ion content was measured using a Varian 730 Inductive Coupled Plasma – Optical Emission Spectrophotometer (ICP-OES) (Agilent Technologies, Santa Clara, CA, United States). The ICP-OES spectrophotometer was calibrated using five multi-element standards containing the ions of interest, at concentrations of 0.2, 0.5, 1, 2, and 5 ppm.

### Statistics

Apart from the microarray and GO enrichment analyses, all statistical analyses were performed using Statistica version 13.3. For one-way ANOVA analyses, significant *p*-values were determined by Fisher LSD *post hoc* analysis. For independent t-tests, Bonferroni multiple testing correction was applied to correct *p*-values.

## Results

### The Salinity Stress Transcriptome Can Be Distinguished From the Osmotic Stress Transcriptome to Identify Salt-Specific Genes

**FIGURE 1:**
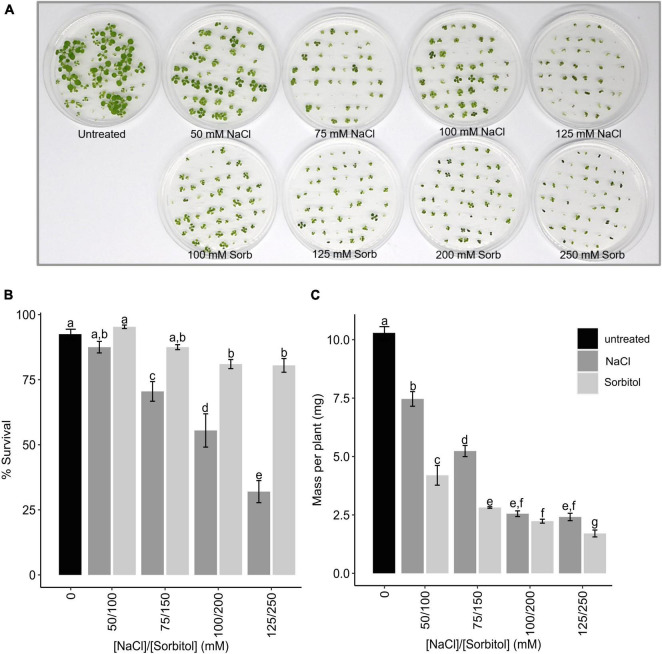
Survival and mass of Arabidopsis Col-0 plants grown on NaCl and sorbitol. Col-0 was germinated and grown for two weeks in petri dishes containing ATS media (0 mM, untreated control) and ATS supplemented with the indicated concentrations of NaCl and sorbitol (Sorb) and survival and plant growth (mass per plant) determined. **(A)** A photograph of the Arabidopsis plants after two weeks growth on each treatment. **(B)** The mean% survival of Col-0 after two weeks growth on each treatment. **(C)** The mean mass per plant of Col-0 after two weeks growth on each treatment. The black bar represents the untreated control, dark gray bars represent NaCl treatments and light gray bars represent sorbitol treatments. The results are an average four petri dishes per treatment with 50 seedling sown per petri dish. Error bars represent standard error. Different letters on the graphs indicate statistically significant differences (*p* ≤ 0.05) in mean% survival/mass per plant, determined by one way ANOVA followed by Fisher LSD *post hoc* analysis.

To investigate how plants cope with the delayed ionic effects of salinity stress (i.e., salt-specific effects), Arabidopsis Col-0 was germinated and grown for two weeks in *Arabidopsis thaliana* salt (ATS) media supplemented with varying concentrations of NaCl (50, 75, 100, and 125 mM) or iso-osmolar concentrations of sorbitol (100, 150, 200, and 250 mM) and microarray analysis was used to profile the transcriptome. This dose range was chosen based on an initial hydroponic experiment in which it was found that Arabidopsis Col-0 could survive when grown for more than two weeks in the presence of 50-125 mM NaCl and could reach maturity when grown in 50-100 mM NaCl. However, plants grown for extended periods on 100 and 125 mM NaCl were severely stunted (Supplementary Figures 1A,B). Sorbitol was chosen to impose an equivalent osmotic stress without the ionic effects of NaCl since Arabidopsis does not take up sorbitol (Klepek et al., 2010). When grown in petri dishes, Arabidopsis survival was inhibited in a dose-dependent manner at NaCl concentrations exceeding 50 mM (Figures 1A,B). Plant growth (measured as mass per plant) was inhibited across the entire dose range, with severe growth inhibition again being observed at NaCl concentrations of 100 mM and higher (Figures 1A,C). Iso-osmolar sorbitol had slightly different effects causing less inhibition of survival but greater inhibition of growth (mass per plant) (Figures 1A-C). Notably, plants on 50 and 75 mM NaCl grew bigger (mass per plant) than plants grown on iso-osmolar sorbitol suggesting that plants can maintain growth under saline conditions to a greater extent than plants grown under osmotic stress (Figure 1C).

**FIGURE 2:**
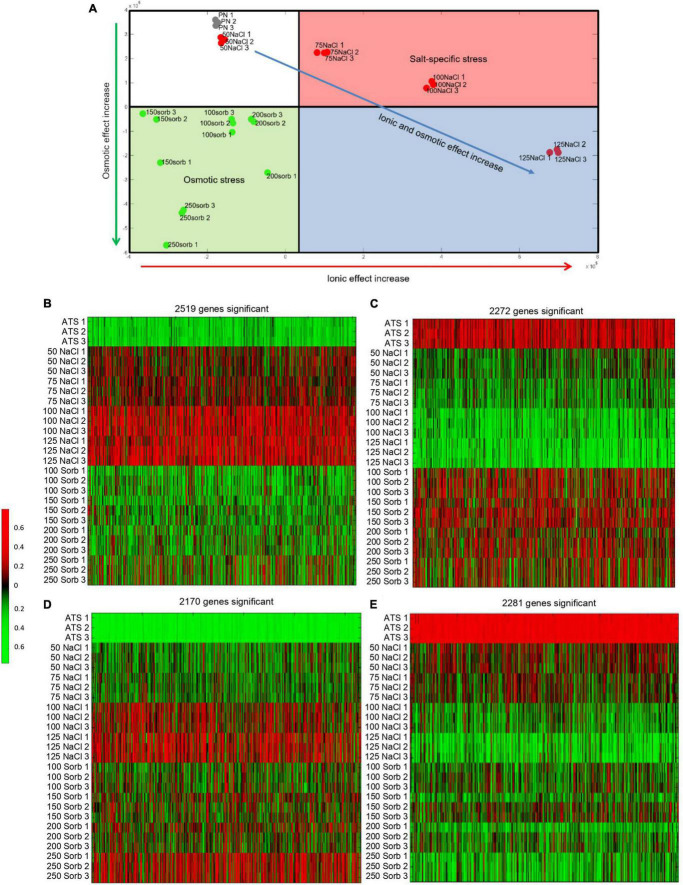
Dimensional reduction plot and heat maps illustrating the genes differentially expressed in plants grown on NaCl and sorbitol. Microarray analysis was performed on plants grown in NaCl or iso-osmolar concentrations of sorbitol for two weeks from germination. The dimensional reduction plot (**A**) was generated from the normalized data to show how the data separated along two principal components corresponding to the ionic effect and osmotic effect of the treatments. The heat maps (**B-E**) were generated from lists of genes that were differentially expressed in plants grown on NaCl but not sorbitol compared to the untreated control (salt-specific genes) and genes that were differentially expressed both in plants grown on NaCl and sorbitol (osmotic genes) compared to the untreated control. **(B):** Expression of 2,519 genes increased significantly (red) in plants grown on NaCl but had different patterns of expression in response to sorbitol compared to the untreated control, **(C):** Expression of 2,272 genes significantly decreased (green) in plants grown on NaCl but had different patterns of expression in plants grown on sorbitol compared to the untreated control, **(D):** Expression of 2,170 genes significantly increased (red) in plants grown on NaCl and sorbitol compared to the untreated control, **(E):** Expression of 2,281 genes significantly decreased (green) in NaCl and sorbitol treatment compared to the untreated control. Genes are represented in columns and control (ATS) or NaCl/sorbitol (Sorb) treatment concentrations in rows. Numbers on the scale bar represent gene expression after quantile normalization, z-score transformation, and logarithmic scaling of the data.

A dimensional reduction analysis of the normalized microarray data separated the data along two principal components that correspond to the ionic and osmotic effects of the treatments (Figure 2A). The data divided into four clusters. The first cluster included the untreated controls (ATS1, ATS2, and ATS3) and the 50 mM NaCl treatments which impose low ionic and osmotic stress on the plant. The second cluster included the 75 and 100 mM NaCl treatments that have a high ionic effect and low osmotic effect. The third cluster included all the sorbitol treatments that exert a high osmotic effect and low ionic effect. The final cluster incorporated the 125 mM NaCl treatments that have both a high ionic and high osmotic component. The minor difference between the control and 50 mM NaCl samples shows that this treatment imposed very little stress on the plant. Clear separation between the 75 mM and 100 mM NaCl treatments and their corresponding iso-osmolar sorbitol concentrations reveals an obvious difference between plants grown under saline conditions compared to those grown under osmotic stress conditions. Furthermore, a dose dependency in the adaptive response to NaCl is apparent. Finally, the separation of the 125 mM NaCl samples along the same component as the sorbitol samples suggests that, at this NaCl dose, there is a substantial osmotic effect. Therefore, the NaCl concentrations tested here are suitable for addressing our biological question since they range from a mild stress at 50 mM to a more severe stress at 125 mM NaCl and there is a clear difference between the transcriptomes of plants grown in NaCl and those grown in iso-osmolar sorbitol.

To understand how plants tolerate salt, the list of genes differentially expressed in plants grown on NaCl was compared to the list of genes differentially expressed in plants grown on sorbitol. To generate each of these differentially expressed gene lists, a Mann-Whitney test was performed to determine whether the distribution of expression data for each gene was different among the treated samples compared to the control samples, yielding a *p*-value for each gene. This was corrected for multiple hypothesis testing according to the method of Benjamini and Hochberg. Finally, the list of genes that were significantly (corrected *p* < 0.01) differentially expressed in plants grown in NaCl was compared to the list of genes that were significantly differentially expressed in plants grown in sorbitol to generate a list of salt-specific genes. These are the genes which respond significantly to NaCl but not to sorbitol. This gave rise to a list of 2,519 genes whose expression increased in a salt-specific manner and 2,272 genes whose expression decreased in a salt-specific manner (Figures 2B,C and Supplementary Table 1). Additionally, the intersection of the NaCl and sorbitol gene lists was determined to generate a list of osmotic genes (genes that are differentially expressed in plants grown on NaCl and in plants grown on sorbitol). This produced a list of 2,170 genes whose expression increased and 2,281 genes whose expression decreased in plants grown under osmotic stress conditions (Figures 2D,E and Supplementary Table 1). To validate the microarray data, the expression of several genes (salt-specific, osmotic and non-responsive) was confirmed by RT-qPCR (Supplementary Figure 2). Interestingly, the salt-specific gene list contains the well known Na^+^ transporters, *SALT OVERLY SENSITIVE 1* (*SOS1*) and the *Na^+^/H^+^ EXCHANGER*, *NHX3* (Supplementary Table 1) which is consistent with our knowledge of ion-dependent mechanisms to tolerate salinity stress (Ji et al., 2013; Bassil et al., 2019) and demonstrates the accuracy of our data and analysis pipeline.

### Salt-Specific Genes Are Enriched in the Functional Term ‘Response to Auxin’

To gain insight into the functional relevance of these gene lists, Gene Ontology (GO) enrichment analyses were performed. The salt-specific and osmotic gene lists were first filtered to retain genes whose expression increased or decreased by two-fold or more in at least one NaCl or sorbitol concentration (Supplementary Table 2). The GO analysis was performed using both the “classic” and “weight.01” (weighted) algorithms in topGO followed by Fisher’s exact tests to identify enriched terms. In brief, while the classic algorithm tests each GO category independently, the weighted algorithm penalizes scores for more general terms that share genes with more specific neighboring terms, weighting the analysis to the identification of more specific and therefore informative ontologies. We additionally restricted our analyses to GO terms that were represented by > 10 significant genes as these are more likely to be functionally important compared to terms that are represented by few genes. The significantly enriched GO terms for biological processes associated with the salt-specific gene list were distinct from those associated with the osmotic gene list (Table 1). As expected, GO terms enriched in the osmotic gene list included “response to water deprivation,” “response to abscisic acid” and “response to salt stress.” Genes associated with these terms included many well-known markers of osmotic stress such as *LATE EMBRYOGENESIS ABUNDANT (LEA)*, *RESPONSE TO DEHYDRATION (RD)*, *STRESS RESPONSIVE (KIN)*, *COLD REGULATED (COR)*, *ABA INSENSITIVE (ABI)* genes and the proline biosynthesis gene, *DELTA1-PYRROLINE-5-CARBOXYLATE SYNTHASE 1 (P5CS1)* (Table 2 and Supplementary Table 3). Furthermore, this suggests that many of the genes annotated with the GO term “response to salt stress” respond to the osmotic component of salinity stress. The salt-specific gene list only produced two enriched GO terms that had more than ten associated genes (Table 1). One of these was the GO term “cellular response to hypoxia” and the other was “response to auxin.” The salt-specific genes annotated to be involved in the “cellular response to hypoxia” include several general defense genes (Supplementary Table 3) and the enrichment in this term could be due to the similarity between the plant response to hypoxia and the response to salinity stress. Both stresses, for example, lead to Ca^2+^ signaling, membrane depolarization and production of reactive oxygen species (Wang et al., 2017). The enrichment in the GO term “response to auxin” is of particular interest because auxin is a major phytohormone which plays an indispensable role during plant growth, and thus might alter plant growth in saline environments. Genes annotated to be involved in the ‘response to auxin’ included many *SMALL AUXIN UPREGULATED RNA* (SAUR) genes, *AUXIN RESISTANT 1* (*AXR1*), *AUXIN-RESPONSIVE GH3 FAMILY PROTEIN* (*GH3.5*) and *PINOID-BINDING PROTEIN 1* (*PBP1*) among others (Table 3). The *SAURs* are a large gene family known to be rapidly induced when auxin concentrations increase (Calderon-Villalobos et al., 2010). Most of the *SAURs* in the salt-specific gene list increased in expression in plants grown under saline conditions (Table 3). This suggests that auxin levels increase in a salt-specific manner which would subsequently induce the expression of the *SAURs*. In contrast, the list of osmotic genes was enriched in GO terms for different hormones including “response to abscisic acid” and “response to jasmonic acid” (Table 1) and no auxin-related GO terms were enriched in this gene list. Thus, it appears that auxin may be specifically involved in adaptation to salinity stress, whereas several other hormones are involved in osmotic stress tolerance. Taken together, the GO enrichment results indicate that (1) there are salt-specific (ion-dependent) responses that can be distinguished from osmotic stress responses as previously reported (Donaldson et al., 2004; Munns and Tester, 2008; Shavrukov, 2013; Julkowska and Testerink, 2015), (2) genes that were previously functionally annotated to respond to salt likely respond to the osmotic component of salinity stress, and (3) auxin potentially plays an important role during growth in saline conditions.

**TABLE 1:** The Gene Ontology (GO) terms enriched in the osmotic and salt-specific gene lists.

GO term	Description	Number in background	Number in gene set	Expected	Fisher classic	Fisher weighted
**Osmotic gene list (1879 genes)**						
GO:0009414	response to water deprivation	398	69	26.94	4.2e-13	2.2e-10
GO:0009611	response to wounding	222	44	15.03	9.3e-11	2.9e-10
GO:0009753	response to jasmonic acid	205	43	13.88	2.2e-11	1.8e-09
GO:0006979	response to oxidative stress	467	68	31.61	1.7e-09	2.4e-09
GO:0071456	cellular response to hypoxia	239	42	16.18	1.1e-08	1.1e-08
GO:0009737	response to abscisic acid	578	88	39.12	5.6e-13	2.6e-08
GO:0080167	response to karrikin	128	26	8.66	3.8e-07	3.8e-07
GO:0006952	defense response	1103	130	74.66	2.8e-10	2.9e-06
GO:0019761	glucosinolate biosynthetic process	52	16	3.52	1.8e-07	4.4e-06
GO:0009625	response to insect	32	11	2.17	4.5e-06	4.5e-06
GO:0009718	anthocyanin-containing compound biosynthesis	33	11	2.23	6.3e-06	1.0e-05
GO:0055114	oxidation-reduction process	754	73	51.04	0.00128	1.5e-05
GO:0009828	plant-type cell wall loosening	37	11	2.5	2.2e-05	2.2e-05
GO:0010114	response to red light	63	16	4.26	3.1e-06	2.5e-05
GO:0009651	response to salt stress	485	58	32.83	1.8e-05	5.9e-05
GO:0071215	cellular response to abscisic acid stimulus	234	36	15.84	3.2e-06	0.00018
GO:2000280	regulation of root development	97	16	6.57	0.00077	0.00041
GO:0009409	response to cold	427	50	28.9	0.00011	0.00053
GO:0019748	secondary metabolic process	363	55	24.57	1.6e-08	0.00072
GO:0010218	response to far red light	44	11	2.98	0.00013	0.00078
**Salt specific gene list (962 genes)**						
GO:0071456	cellular response to hypoxia	239	27	8.15	5.5e-08	5.5e-08
GO:0009733	response to auxin	337	25	11.5	0.00025	1.4e-05

*“Number in background” is the number of genes in the Arabidopsis genome with the associated GO term, “Number in gene set” is the number of genes in the query gene list with the associated GO term, “Expected” is the number of genes with the associated GO term expected to appear in the query list, “Fisher classic” represents the p-value after a Fisher classic statistical test for significance of enrichment, “Fisher weighted” represents the p-value after a weighted Fisher statistical test for significance of enrichment.*

**TABLE 2:** The genes from the osmotic gene list annotated with the enriched GO term “response to salt stress.”

Gene accession number	Gene name/description	log2 fold change in response to NaCl	log2 fold change in response to sorbitol
AT1G07400	*HEAT SHOCK PROTEIN 17.8 (HSP17.8)*	1.1	1.3
AT1G16850	uncharacterized transmembrane protein	3.2	3.1
AT1G50960	*GIBBERELLIN 2-OXIDASE 7 (GA2OX7)*	3.1	1.5
AT1G52400	*BETA GLUCOSIDASE 18 (BGLU18)*	3.0	1.1
AT1G56060	*CYSTEINE-RICH TRANSMEMBRANE MODULE 3 (CYSTM3)*	2.2	1.5
AT1G56600	*GALACTINOL SYNTHASE 2 (GOLS2)*	3.1	4.1
AT1G61340	*F-BOX STRESS INDUCED 1 (FBS1)*	1.0	0.5
AT1G65500	*SECRETED TRANSMEMBRANE PEPTIDE 6 (STMP6)*	3.1	3.7
AT2G29500	*HEAT SHOCK PROTEIN 17.6B (HSP17.6B)*	1.4	2.1
AT2G33380	*CALEOSIN 3 (CLO-3)*	3.6	3.0
AT2G35612	uncharacterized copper amine oxidase family protein	2.4	3.8
AT2G36270	*ABA INSENSITIVE 5 (ABI5)*	1.2	2.0
AT2G37760	*ALDO-KETO REDUCTASE FAMILY 4 MEMBER C8 (AKR4C8)*	1.9	1.0
AT2G38170	*CATION EXCHANGER 1 (CAX1)*	1.3	0.7
AT2G39800	*DELTA1-PYRROLINE-5-CARBOXYLATE SYNTHASE 1 (P5CS1)*	2.7	3.5
AT2G41010	*CALMODULIN (CAM)-BINDING PROTEIN OF 25 (CAMBP25)*	1.1	0.2
AT2G41870	*REMORIN GROUP 4.2 (REM4.2)*	0.8	1.7
AT2G42540	*COLD-REGULATED 15A (COR15A)*	4.4	5.1
AT2G47770	*TSPO(OUTER MEMBRANE TRYPTOPHAN-RICH SENSORY PROTEIN)-RELATED (TSPO)*	5.1	7.0
AT3G49580	*RESPONSE TO LOW SULFUR 1 (LSU1)*	2.9	2.1
AT3G63060	*EID1-LIKE 3 (EDL3)*	1.6	1.2
AT4G11890	*ABA- AND OSMOTIC-STRESS-INDUCIBLE RECEPTOR-LIKE CYTOSOLIC KINASE1 (ARCK1)*	1.3	1.9
AT4G12480	*EARLY ARABIDOPSIS ALUMINUM INDUCED 1 (EARLI 1)*	3.2	2.5
AT4G19810	*CLASS V CHITINASE (CHIC)*	1.7	1.3
AT4G23600	*JASMONIC ACID RESPONSIVE 2 (JR2)*	2.5	2.3
AT4G34710	*ARGININE DECARBOXYLASE 2 (ADC2)*	1.3	1.4
AT5G02020	*SALT INDUCED SERINE RICH (SIS)*	2.0	1.7
AT5G24090	*CHITINASE A (CHIA)*	1.3	1.5
AT5G24770	*VEGETATIVE STORAGE PROTEIN 2 (VSP2)*	3.1	2.8
AT5G25610	*RESPONSIVE TO DESICCATION 22 (RD22)*	1.5	1.5
AT5G28510	*BETA GLUCOSIDASE 24 (BGLU24)*	2.8	1.8
AT5G46830	*NACL-INDUCIBLE GENE 1 (NIG1)*	2.8	1.7
AT5G50720	*HVA22 HOMOLOG E (HVA22E)*	0.9	1.2
AT5G52300	*LOW-TEMPERATURE-INDUCED 65 (LTI65)*	4.8	7.5
AT5G52310	*COLD REGULATED 78 (COR78)*	2.4	2.6
AT5G59310	*LIPID TRANSFER PROTEIN 4 (LTP4)*	4.4	6.0
AT5G62490	*HVA22 HOMOLOG B (HVA22B)*	3.4	2.3
AT5G62520	*SIMILAR TO RCD ONE 5 (SRO5)*	1.0	0.7
AT1G01620	*PLASMA MEMBRANE INTRINSIC PROTEIN 1;3 (PIP1;3)*	−1.1	−0.7
AT1G05680	*URIDINE DIPHOSPHATE GLYCOSYLTRANSFERASE 74E2 (UGT74E2)*	−2.0	−1.3
AT1G35910	*TREHALOSE-6-PHOSPHATE PHOSPHATASE D (TPPD)*	−2.3	−2.1
AT1G43160	*ETHYLENE RESPONSIVE FACTOR113 (RAP2.6)*	−1.7	−1.2
AT1G47840	*HEXOKINASE 3 (HXK3)*	−1.4	−1.2
AT1G70170	*MATRIX METALLOPROTEINASE (MMP)*	−0.8	−1.7
AT2G01900	*INOSITOL POLYPHOSPHATE PHOSPHATIDYLINOSITOL 5-PHOSPHATASE9 (T5PTASE9)*	−1.9	−1.7
AT2G02820	*MYB DOMAIN PROTEIN 88 (MYB88)*	−1.2	−0.5
AT2G15390	*FUCOSYLTRANSFERASE 4 (FUT4)*	−1.3	−0.7
AT2G47460	*MYB DOMAIN PROTEIN 12 (MYB12)*	−1.1	−0.8
AT3G02140	*ABI FIVE BINDING PROTEIN 4 (AFP4)*	−1.8	−1.4
AT3G50310	*ABA-INSENSITIVE PROTEIN KINASE 1 (AIK1)*	−2.1	−1.7
AT3G54770	*ABA-REGULATED RNA-BINDING PROTEIN 1 (ARP1)*	−1.6	−1.0
AT4G20260	*PLASMA-MEMBRANE ASSOCIATED CATION-BINDING PROTEIN 1 (PCAP1)*	−0.3	−1.1
AT5G17960	uncharacterized Cysteine/Histidine-rich C1 domain family protein	−3.5	−1.4
AT5G24120	*SIGMA FACTOR 5 (SIG5)*	−0.8	−1.2
AT5G44610	*PLASMA MEMBRANE ASSOCIATED CA2* + *-BINDING PROTEIN-2 (PCAP2)*	−1.4	−0.9
AT5G49630	*AMINO ACID PERMEASE 6 (AAP6)*	−1.1	−1.3
AT5G63650	*SNF1-RELATED PROTEIN KINASE 2.5 (SNRK2.5)*	−1.5	−1.0

*Information on the “Gene name/description” associated with each gene accession number is reported according to TAIR (https://www.arabidopsis.org/). The values in the “log2 fold change in response to NaCl” column are the greatest fold change seen for each gene across the four NaCl treatments. The values in the “log2 fold change in response to sorbitol” are the greatest fold change seen for each gene across the four sorbitol treatments. Osmotic genes are significantly differentially expressed in response to NaCl and sorbitol treatments. The log2 fold change values are relative to the untreated control.*

**TABLE 3:** The genes from the salt-specific gene list annotated with the enriched GO term “response to auxin stimulus.”

Gene accession number	Gene name/description	Log2 fold change in response to NaCl
AT5G53590	*SMALL AUXIN UPREGULATED RNA 30 (SAUR30)*	1.28
AT5G37770	*CALMODULIN-LIKE 24 (CML24)*	1.41
AT5G27780	*SMALL AUXIN UPREGULATED RNA 75 (SAUR75)*	3.06
AT4G38860	*SMALL AUXIN UPREGULATED RNA 16 (SAUR16)*	2.52
AT4G38850	*SMALL AUXIN UPREGULATED RNA 15 (SAUR15)*	1.58
AT4G38840	*SMALL AUXIN UPREGULATED RNA 14 (SAUR14)*	1.52
AT4G36110	*SMALL AUXIN UPREGULATED RNA 9 (SAUR9)*	1.44
AT4G19690	*IRON-REGULATED TRANSPORTER 1 (IRT1)*	3.35
AT3G28910	*MYB DOMAIN PROTEIN 30 (MYB30)*	1.21
AT3G03840	*SMALL AUXIN UPREGULATED RNA 27 (SAUR27)*	3.06
AT3G03830	*SMALL AUXIN UPREGULATED RNA 28 (SAUR28)*	2.33
AT2G21210	*SMALL AUXIN UPREGULATED RNA 6 (SAUR6)*	1.07
AT1G29460	*SMALL AUXIN UPREGULATED RNA 65 (SAUR65)*	1.29
AT5G54490	*PINOID-BINDING PROTEIN 1 (PBP1)*	–1.32
AT5G47370	*HOMEOBOX ARABIDOPSIS THALIANA (HAT2)*	–1.06
AT5G03310	*SMALL AUXIN UPREGULATED RNA 44 (SAUR44)*	–1.91
AT4G32810	*CAROTENOID CLEAVAGE DIOXYGENASE 8 (CCD8)*	–1.63
AT4G27260	*AUXIN-RESPONSIVE GH3 FAMILY PROTEIN 5 (GH3.5)*	–0.44
AT3G55120	*TRANSPARENT TESTA 5 (TT5)*	–1.15
AT3G12955	*SMALL AUXIN UPREGULATED RNA 74 (SAUR74)*	–1.04
AT3G01220	*HOMEOBOX PROTEIN 20 (ATHB20)*	–1.10
AT1G43040	*SMALL AUXIN UPREGULATED RNA 58 (SAUR58)*	–1.06
AT1G27740	*ROOT HAIR DEFECTIVE 6-LIKE 4 (RSL4)*	–1.39
AT1G13670	*BIG GRAIN LIKE 2 (BGL2)*	–1.56

*Information on the “Gene name/description” associated with each gene accession number is reported according to TAIR (https://www.arabidopsis.org/). The values in the “log2 fold change in response to NaCl” column are the greatest fold change seen for each gene across the four NaCl treatments. Salt-specific genes are significantly differentially expressed in response to NaCl but not sorbitol, hence no log2 fold change in response to sorbitol values are included. The log2 fold change values are relative to the untreated control.*

### Salt-Specific Increases in Indole-3-Acetic Acid Are Driven by Indole-3-Acetic Acid Biosynthesis

**FIGURE 3:**
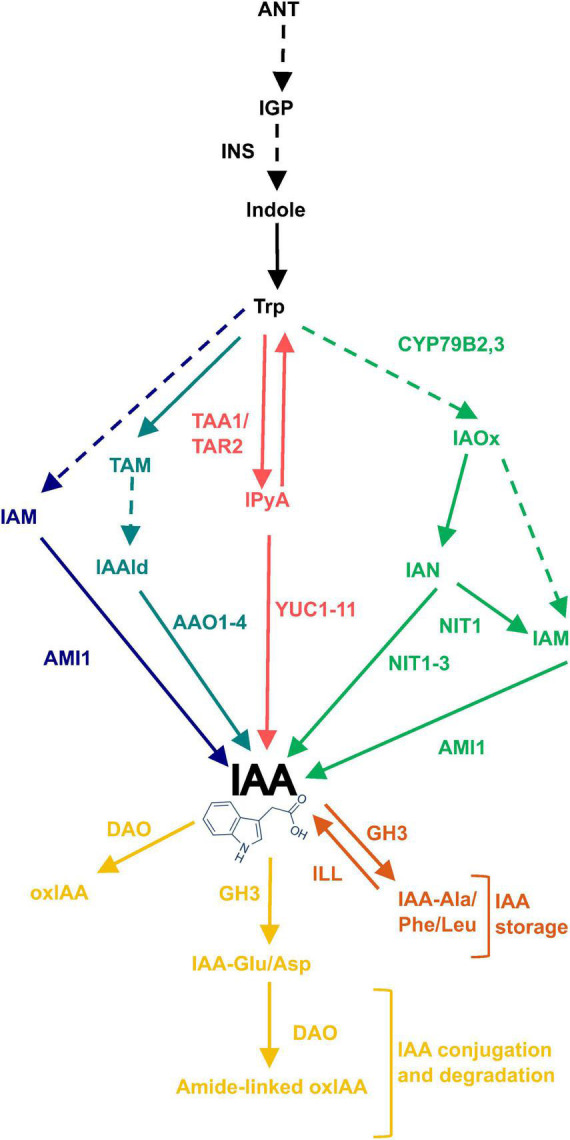
Routes of tryptophan-dependent (TD) IAA biosynthesis and IAA oxidation and conjugation. The TD pathway of IAA biosynthesis has four proposed routes named for their key intermediates: IAM, TAM, IPyA, and IAOx shown in dark blue, light blue, red and green, respectively. Excess free IAA is removed either by direct oxidation or irreversible conjugation to Glu or Asp amino acids which tag it for oxidation, shown in yellow. Alternatively, excess IAA can be conjugated to Ala, Phe or Leu amino acids which tag it for storage, shown in orange. Solid arrows refer to reactions with identified enzymes and dashed arrows refer to unidentified/unconfirmed reactions. Enzymes involved are written next to the relevant arrow. **ANT**, anthranilate; **IGP**, indole-3-glycerol phosphate; **INS**, INDOLE SYNTHASE; **IAOx**, indole-3-acetaldoxime; **CYP79B2,3**, CYTOCHROME P450 FAMILY 79B2,3; **IAN**, indole-3-acetonitrile; **IAM**, indole-3-acetamide; **NIT1-3**, NITRILASE 1-3; **AMI1**, AMIDASE 1; **TAA1**, TRYPTOPHAN AMINOTRANSFERASE OF ARABIDOPSIS 1; **TAR2**, TRYPTOPHAN AMINOTRANSFERASE RELATED 2; **IPyA**, indole-3-pyruvic acid; **YUC1-11**, YUCCA 1-11; **TAM**, tryptamine; **IAAld**, indole-3-acetaldehyde; **AAO1-4**, ARABIDOPSIS ALDEHYDE OXIDASE 1-4; **IAM**, indole-3-acetamide; **oxIAA**, 2-oxoindole-3-acetic acid; **DAO**, DIOXYGENASE FOR AUXIN DEGRADATION.

**FIGURE 4:**
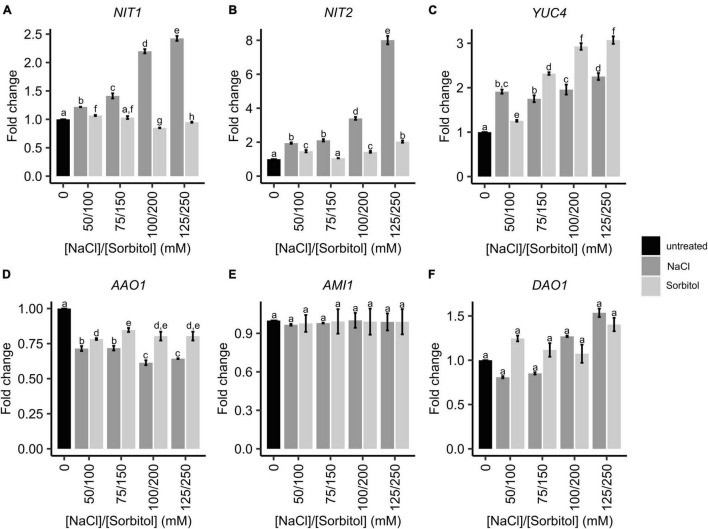
The expression of genes involved in IAA biosynthesis and degradation in Arabidopsis plants grown on NaCl and sorbitol. Arabidopsis Col-0 was germinated and grown for two weeks in petri dishes containing ATS media (0 mM, untreated control) and ATS supplemented with the indicated concentrations of NaCl and sorbitol. Gene expression was determined by microarray analysis. IAA biosynthetic genes include: *NIT1* **(A)**, *NIT2* **(B)**, *YUC4* **(C)**, *AAO1* **(D)** and *AMI1* **(E)**. IAA degradative genes include *DAO1* **(F)**. The black bar represents the untreated control, dark grey bars represent NaCl treatments and light grey bars represent sorbitol treatments. All microarray results are shown as the mean fold change relative to the untreated control (0 mM). The average was calculated from three biological repeats. Error bars represent standard error. Different letters on the graphs indicate statistically significant differences (*p* ≤ 0.05) in mean fold change as determined by one way ANOVA followed by Fisher LSD post-hoc analysis. ***NIT2***: Nitrilase 2, ***NIT1***: Nitrilase 1, ***YUC4***: Flavin monooxygenase-like enzyme 4, ***AAO1***: Arabidopsis aldehyde oxidase 1, ***AMI1***: Indole-3-acetamide 1, ***DAO1***: Dioxygenase for Auxin degradation 1.

The GO analysis hinted that auxin levels may be elevated in plants grown under saline conditions, leading to the increased expression of the auxin-inducible *SAUR* genes. Therefore, the expression of genes that could affect auxin levels, was further investigated in the microarray data. This included genes involved in the tryptophan-dependent IAA biosynthesis pathway and IAA degradation (Figure 3). The expression of *FLAVIN MONOOXYGENASE-LIKE ENZYME 4* (*YUC4*), *NIT1* and *NIT2* genes increased in plants grown on salt and had significantly different expression patterns in plants grown on sorbitol, hence were found on the salt-specific gene list. These genes encode enzymes that catalyze the final steps of the IPyA and IAOx branches of TD IAA biosynthesis, respectively. In particular, the expression of *NIT1*, *NIT2*, and *YUC4* increased significantly in response to all NaCl treatments compared to the untreated control (Figures 4A-C). Additionally, the expression of *NIT1* and *NIT2* was NaCl-dose dependent. *NIT1* and *NIT2* expression did not change significantly in plants grown under any of the sorbitol treatments compared to the untreated control; whereas *YUC4* expression increased significantly in all sorbitol treatments (although its expression in the NaCl treatments was significantly different to the respective iso-osmolar sorbitol treatments). Importantly, *NIT2* showed the greatest change in expression in plants grown under saline conditions compared to the other IAA biosynthetic genes, reaching a maximum of eight-fold induction in 125 mM NaCl (Figure 4B). This was confirmed by RT-qPCR (Supplementary Figure 2). The only IAA biosynthetic gene to be significantly downregulated in plants grown in NaCl and sorbitol, compared to the untreated control, was *ARABIDOPSIS ALDEHYDE OXIDASE 1* (*AAO1*) (Figure 4D). The expression of *INDOLE-3-ACETAMIDE* (*AMI1*) showed no statistically significant difference in expression under any of the treatments compared to the untreated control (Figure 4E). Other genes higher up in the TD IAA biosynthesis pathway, such as *CYTOCHROME P450 FAMILY 79B2,3* (*CYP79B2,3*), did not show a significant change in expression in the transcriptome data (data not shown). Finally, expression of *DIOXYGENASE FOR AUXIN DEGRADATION* (*DAO*), which is involved in IAA degradation, did not change significantly in plants grown in NaCl, suggesting that, under saline conditions, IAA levels are not modulated *via* degradation (Figure 4F).

**FIGURE 5:**
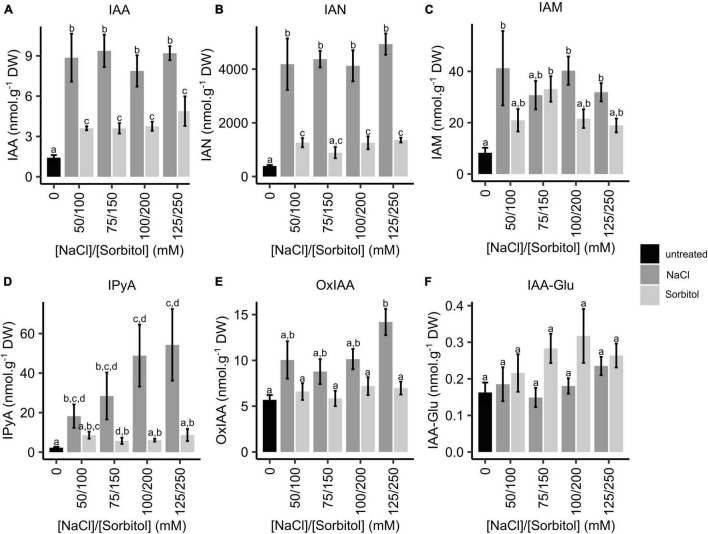
Quantification of IAA, IAA biosynthetic intermediates and IAA oxidation and conjugation products in Arabidopsis plants grown on NaCl and sorbitol. Arabidopsis Col-0 was germinated and grown for two weeks in petri dishes containing ATS media (0 mM, untreated control) and ATS supplemented with the indicated concentrations of NaCl and sorbitol. LC-MS/MS was used to quantify IAA **(A)**, IAN **(B)**, IAM **(C)**, IPyA **(D)**, oxIAA **(E)** and IAA-Glu **(F)**. The black bar represents the untreated control, dark grey bars represent NaCl treatments and light grey bars represent sorbitol treatments. The results are the mean of five biological repeats. Error bars represent standard error. Different letters on the graphs indicate statistically significant differences (*p* ≤ 0.05) in metabolite concentration (nmol.g^–1^ DW) as determined by one way ANOVA followed by Fisher LSD post-hoc analysis. **IAA**: indole-3-acetic acid, **IAN**: indole-3-acetonitrile, **IAM**: indole-3-acetamide, **IPyA**: indole-3-pyruvic acid, **oxIAA**: oxidised IAA, **IAA-Glu**: glutamate irreversibly conjugated to IAA.

To determine whether changes in the expression of IAA biosynthetic genes are reflected by changes in IAA levels in plants grown under saline or osmotic stress conditions, LC-MS/MS analysis of IAA and its metabolites was performed. Whole seedlings were grown for two weeks in petri dishes containing ATS media (control) or ATS media supplemented with varying concentrations of NaCl (50, 75, 100, and 125 mM) or iso-osmolar concentrations of sorbitol (100, 150, 200, and 250 mM). This experimental set-up was identical to the microarray experiment. While IAA concentrations increased significantly in plants grown on both NaCl and sorbitol, the magnitude of the increase was significantly greater in plants grown on NaCl, with a maximum increase of 6.6-fold in 75 mM NaCl, whereas the maximum increase in any of the sorbitol treatments was 3.4-fold (Figure 5A). Interestingly, the concentration of IAA observed in plants grown on NaCl was not dose-dependent, with no significant increase observed from 50 to 125 mM NaCl. The concentrations of the IAA biosynthetic intermediates, indole-3-acetonitrile (IAN), indole-3-acetamide (IAM) and indole-3-pyruvic acid (IPyA), were higher in plants grown under saline conditions compared to the untreated controls (Figures 5B-D). The increase in IAM levels was modest and there was no significant difference in IAM levels in plants grown on NaCl compared to iso-osmolar sorbitol (Figure 5C). The levels of IPyA displayed a salt-specific and dose-dependent increase (Figure 5D). Notably, changes in IAN (the substrate for NIT2) levels in plants grown in NaCl and sorbitol mirror the observed changes in IAA (Figures 5A,B). The products of IAA oxidation and conjugation (oxIAA and IAA-Glu) were also analyzed to determine whether IAA is conjugated and/or degraded in plants grown on NaCl or sorbitol. The concentrations of these metabolites did not change significantly under any of the NaCl or sorbitol treatments compared to the untreated control (Figures 5E, F) which agrees with the *DAO1* gene expression results (Figure 4F). Auxin levels are much higher in plants grown under saline conditions compared to plants grown in osmotic stress. However, the increased expression of the auxin biosynthesis genes, *NIT1*, *NIT2*, and *YUC4* does not perfectly mimic the observed increases in auxin and the upstream metabolites, IAN and IPyA, suggesting that there may be additional posttranscriptional regulation of these (or other) enzymes to modulate auxin levels. Nevertheless, the strong salt-specific induction of *NIT2* could be responsible, at least in part, for the increased IAA concentrations observed in plants grown under saline conditions compared to those grown under osmotic stress.

### Overexpression of *Nitrilase 2* Increases IAA Levels and Improves Salt Tolerance

To assess whether NIT2 is functionally important in plant growth under saline conditions, Arabidopsis lines with altered *NIT2* expression were analyzed to determine the contribution of NIT2 to IAA biosynthesis and the phenotype of plants grown under saline and osmotic stress conditions. Salt tolerance phenotyping was performed on two independent homozygous T-DNA mutant lines (*nit2-1* and *nit2-2*) which have reduced *NIT2* expression (Supplementary Figure 3) and no difference in salt tolerance was observed when compared to the Col-0 wildtype (Supplementary Figures 4A-D). Subsequently, a previously characterized (Lehmann et al., 2017) *nit2*-RNAi line which has knocked down expression of all three *NIT-1* family genes was phenotyped and again there was no observed difference in salt tolerance (Supplementary Figures 4E,F).

**FIGURE 6:**
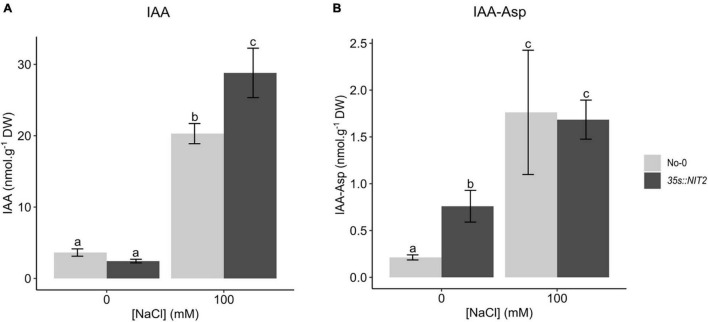
Quantification of IAA and IAA-Asp in No-0 and *35s:NIT2* plants grown on NaCl. No-0 (wildtype) and *35s:NIT2* were germinated and grown for two weeks in petri dishes containing ATS media (0 mM, untreated control) and ATS supplemented with 100 mM NaCl. IAA (**A**) and IAA-Asp (**B**) were quantified by LC-MS/MS. The light gray bars represent No-0 and dark gray bars represent *35s:NIT2*. The results are the mean of five biological repeats. Error bars represent standard error. Different letters on the graph indicate statistically significant differences (*p* ≤ 0.05) in metabolite concentration (nmol.g^–1^ DW) as determined by one way ANOVA followed by Fisher LSD *post hoc* analysis.

In parallel, a previously characterized *NIT2* overexpressing line (*35s:NIT2*; Normanly et al., 1997) was investigated for its function in plant growth under saline conditions. In untreated control conditions, the *35s:NIT2* line showed an increase in *NIT2* expression of more than 17 000-fold compared to the genetic background, No-0 (Supplementary Figure 5), confirming the genotype of this line. To determine whether overexpression of *NIT2* alters IAA levels *in planta*, LC-MS/MS analysis was performed on *35s:NIT2* and No-0 seedlings grown for two weeks in petri dishes containing ATS media (control) or ATS media supplemented with 100 mM NaCl. Surprisingly, the concentration of IAA in plants grown under control conditions did not differ between No-0 and *35s:NIT2* (Figure 6A). In contrast, in plants grown on 100 mM NaCl, the concentration of IAA increased significantly compared to control conditions for both plant lines but IAA levels were significantly greater in *35s:NIT2* plants grown on NaCl compared to No-0. Thus, *35s:NIT2* produces more IAA than the wild type, but only under saline conditions. To investigate why there was no increase in IAA in the *35s:NIT2* line (compared to No-0) in untreated control conditions, IAA-Asp, a product of IAA conjugation that targets IAA for degradation, was measured. In plants grown under control conditions the concentration of IAA-Asp was significantly greater in *35s:NIT2* plants compared to No-0 whereas there was no difference in IAA-Asp levels between No-0 and *35s:NIT2* grown in 100 mM NaCl (Figure 6B). This could indicate that excess IAA produced in the *35s:NIT2* overexpression line during growth in standard conditions is removed *via* degradation through IAA-Asp conjugation. While IAA-Asp levels are elevated in both *35s:NIT2* and No-0 plants grown under saline conditions, there is no significant difference in IAA-Asp between these lines. Since IAA levels are elevated in the *35s:NIT2* line under saline conditions (compared to No-0) this suggests that the excess IAA produced by the *35s:NIT2* line during growth in saline conditions is beneficial and thus does not require additional degradation as seen in the untreated control conditions.

**FIGURE 7:**
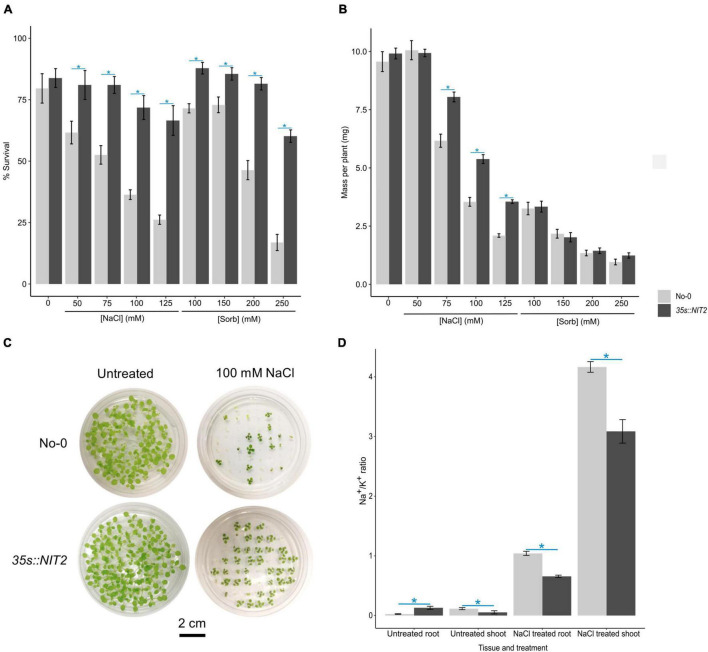
Functional characterization of the Arabidopsis *NIT2* overexpression line grown on NaCl and sorbitol. For **(A**-**C)**, No-0 (wildtype) and *35s:NIT2* were germinated and grown for two weeks in petri dishes containing ATS media (0 mM, untreated control) and ATS supplemented with the indicated concentrations of NaCl and sorbitol (Sorb) and survival and plant growth (mass per plant) determined. The results are an average of three independent experiments. **(A):** The average% survival of No-0 and *35s:NIT2* in untreated, NaCl treated or sorbitol treated conditions. **(B):** The average mass per plant of No-0 and *35s:NIT2* in untreated, NaCl treated or sorbitol treated conditions. **(C):** A photograph of No-0 and *35s:NIT2* after two weeks growth on petri dishes containing ATS media and ATS supplemented with 100 mM NaCl. For **(D),** No-0 and 35s:*NIT2* were grown hydroponically for three weeks on 1/4 strength ATS media then transferred onto 1/4 strength ATS media supplemented with 0 mM or 75 mM NaCl and grown for one additional week. The Na^+^ and K^+^ content in untreated and NaCl treated shoot and root tissue was determined *via* ICP-OES analysis and the Na^+^/K^+^ ratio calculated. The results are the mean of six biological repeats. For all figures, the light gray bars represent No-0 and dark gray bars represent *35s:NIT2*. Error bars represent standard error. A star indicates a statistically significant difference in mean between No-0 and 35s:*NIT2* in that treatment/tissue. Significance was determined by independent t-tests with a corrected *p*-value ≤ 0.05 considered significant. The Bonferroni method was used to correct *p*-values for multiple testing.

To determine whether *NIT2* overexpression impacts salt tolerance, the survival and growth (mass per plant) of *35s:NIT2* and No-0 were compared after two weeks growth on untreated control media or media supplemented with varying concentrations of NaCl or iso-osmolar sorbitol. The survival of *35s:NIT2* was significantly greater than No-0 in all NaCl and sorbitol treatments (Figures 7A,C), with the survival of the *35s:NIT2* plant line only decreasing (compared to the ATS control) in the highest NaCl and sorbitol concentrations (100 mM and 125 mM NaCl and 250 mM sorbitol). Salt treatment significantly reduced the growth of both plant lines but the average mass per plant for the *35s:NIT2* line was significantly greater than No-0 when plants were grown on 75, 100, and 125 mM NaCl (Figures 7B,C). Growth was significantly inhibited by all sorbitol treatments compared to the untreated control in both the wild type and *NIT2* overexpression line, with no differences observed between the mass per plant for both plant lines at any sorbitol concentration. Overall, growth of the *NIT2* overexpression line was less inhibited by NaCl compared to No-0 but was not different from No-0 in its response to sorbitol. To investigate whether the improved growth of the *35s:NIT2* line under saline conditions is the result of an improved ability to tolerate ionic stress, Na^+^ and K^+^ content was measured in the root and shoot tissue of *35s:NIT2* and No-0 plants grown under untreated control and saline conditions. Notably, the *35s:NIT2* line was better able to prevent Na^+^ ions from accumulating in the sensitive shoot tissue under saline conditions, compared to No-0 (Supplementary figure 6A). Furthermore, the *35s:NIT2* line was better able to prevent detrimental K^+^ loss from the root under saline conditions, compared to No-0 (Supplementary Figure 6B). This culminated in the *35s:NIT2* line having an improved (lower) Na:K ratio in both the root and the shoot tissue of plants grown under saline conditions, compared to No-0 (Figure 7D). Thus, the *NIT2* overexpression line has higher IAA levels and improved growth, specifically in NaCl, suggesting that NIT2 could be functionally important for producing auxin and maintaining ion homeostasis and growth under saline conditions, and plant salt tolerance.

## Discussion

Previous salt transcriptome experiments have not succeeded in identifying salt tolerance genes that have been manipulated to develop transgenic salt tolerant crops. In such experiments, plants were typically shock treated with high concentrations of NaCl for short periods of time that did not allow Na^+^ and Cl^–^ ions to accumulate to toxic levels. Thus, genes identified in these experiments were most likely osmotic stress genes (Shavrukov, 2013). Manipulation of such osmotic stress genes may improve salt tolerance but could interfere with the plant response to other stresses, since osmotic stress is also caused by drought, low temperature, and pathogens. The limited successes that have been realized in the development of salt tolerant crops have come about through manipulation of Na^+^ transporters that are salt-specific (Munns et al., 2012). For future food security, the development of salt tolerant crops, with enhanced productivity on increasingly saline soils, requires the identification of salt-specific genes.

Our experimental design allowed us to differentiate between the osmotic and ionic components of salinity stress and identify salt-specific genes (Figure 2). The osmotic gene list was enriched in GO terms and genes that are well known to be important for the plant response to osmotic stress, including “response to water deprivation” and “response to ABA.” However, the GO term “response to salt stress”’ was enriched in the osmotic gene list rather than the salt-specific gene list, supporting the argument of Shavrukov (2013) that previous salt transcriptome experiments have identified osmotic stress genes rather than salt-specific genes (Tables 1, 2). In our salt-specific gene list we identified genes known to be involved in ion-dependent mechanisms of salt tolerance, for example *SOS1* and *NHX3* (Supplementary Table 1), validating our experimental design. Unexpectedly, the salt-specific gene list was enriched in the GO term “response to auxin.” This suggests that auxin modulates salt-specific adaptations to salinity stress whereas other hormones are required for plants to cope with the osmotic component of salinity stress (Table 1). This was surprising as it is often the case that hormones act together, whether agonistically or antagonistically, to control the balance between growth and stress responses (Ryu and Cho, 2015; Verma et al., 2016; Berens et al., 2017; Ku et al., 2018; Yang et al., 2019). The salt-specific genes associated with the GO term “response to auxin” are known to be rapidly induced in response to auxin (Table 3). Thus, we investigated auxin levels in plants grown under saline and osmotic stress conditions and found that IAA concentrations are elevated in plants grown on NaCl to a greater degree than in plants grown on iso-osmolar concentrations of sorbitol (Figure 5).

Several other studies have analyzed IAA modulation of plant growth in response to NaCl by measuring the effects of exogenous IAA application (Iqbal and Ashraf, 2007; Jung and Park, 2011; Naser and Shani, 2016), altering IAA-related gene expression (Jiang and Deyholos, 2006; Iglesias et al., 2014), directly measuring IAA concentrations (Prakash and Prathapasenan, 1990; Liu et al., 2015; Prerostova et al., 2017; Pavlović et al., 2018) and indirectly analyzing IAA concentration and localization through reporter lines (Wang et al. 2009; 2019; Zolla et al., 2010). However, these studies have been performed in different plant species and have produced contradictory or inconsistent results, with some indicating increases in IAA concentrations and/or signaling during growth in saline environments (Jiang and Deyholos, 2006; Iqbal and Ashraf, 2007; Iglesias et al., 2014; Pavlović et al., 2018; Wang et al., 2019) whilst others reported the opposite (Prakash and Prathapasenan, 1990; Jung and Park, 2011; Liu et al., 2015; Prerostova et al., 2017). Here we used LC-MS/MS, a precise method of metabolite quantification, to measure IAA and IAA metabolite levels in Arabidopsis plants (Novák et al., 2012). Not only did we find that IAA levels are elevated in plants grown under saline conditions, but the concentrations of the upstream intermediates, IAN, IAM and IPyA, were also higher in plants grown in NaCl compared to plants grown in iso-osmolar sorbitol (Figure 5). This indicates that IAA levels are most likely increased through enhanced IAA biosynthesis rather than through changes in IAA conjugation, degradation, or localization, which agrees with the expression data for the IAA biosynthesis genes (Figure 4).

We note that IAA levels are also elevated in plants grown under osmotic stress conditions, as are some of the upstream intermediates and the expression of IAA biosynthesis genes (Figures 4, 5). Nevertheless, there is clearly an additional salt-specific contribution to auxin biosynthesis to elevate auxin levels above those seen in plants grown under osmotic stress. We would argue that this contribution is made by increased auxin biosynthesis *via* NIT2 as this gene is strongly and specifically induced in plants grown under saline conditions (Figure 4 and Supplementary Figure 2) and IAN (the substrate of NIT2) levels closely mirror those of IAA (Figures 5A,B). There has been some dispute in the literature as to whether NIT2 does play a role in auxin biosynthesis, with a report that IAN was not the preferred substrate for NIT2 in recombinant protein assays (Vorwerk et al., 2001). However, several *in planta* studies have provided evidence that NIT2 does synthesize auxin from IAN (Bartling et al., 1992; Normanly et al., 1997; Grsic et al., 1998; Lehmann et al., 2017).

The fact that the increase in *NIT2* expression is NaCl dose-dependent (Figure 4) while IAN and IAA levels are not (Figure 5), points to a more complicated regulation of *NIT2*, potentially at the level of the protein *via* posttranslational modification (PTM). This might explain why the recombinant protein did not show IAN hydrolyzing activity in the *in vitro* assays (Vorwerk et al., 2001) and why constitutive *NIT2* overexpression (Supplementary Figure 5) does not lead to constitutively higher IAA levels under the standard growth conditions used in our study (Figure 6). Indeed, there is evidence that other plant Nitrilases are regulated at the level of PTM. The Nitrilase gene family in Arabidopsis contains *NIT4* and the *NIT1* subfamily, made up of *NIT1*, *NIT2* and *NIT3*, which have high sequence similarity and redundant functions in IAA biosynthesis (Bartling et al., 1992; Bartel and Fink, 1995; Schmidt et al., 1996; Normanly et al., 1997; Vorwerk et al., 2001; Jenrich et al., 2007; Lehmann et al., 2017). The NIT1 and NIT4 proteins have been proposed to undergo PTMs to modulate their activity (Piotrowski et al., 2001; Cutler and Somerville, 2005; Lehmann et al., 2017) and a recent study reported that plant nitrilase substrate specificity is affected by the proteins’ helical twist which is modulated post-translationally (Woodward et al., 2018). Additionally, Lehmann et al. (2017), in their characterization of *NIT1* during auxin-mediated root growth, suggested the need for PTM of NIT1 to alter substrate affinity in a tissue specific manner. NIT1 has been shown to undergo spontaneous *S*-Glutathionylation, a stress-induced, redox-sensitive and reversible modification of cysteine residues which directly regulates protein activity by altering the conformation of the active site (Dixon et al., 2005). However, it is still unclear whether this modification increases or decreases NIT1 enzyme activity. Lastly, a GFP-NIT1 fusion protein displayed rapid aggregation in response to wounding and herbicide-induced cell death in Arabidopsis (Cutler and Somerville, 2005). Although not tested, NIT1 aggregation could modulate protein activity in response to stress. It is conceivable that similar modifications to the NIT2 protein under saline conditions could be important for substrate binding and enzyme activity.

The *NIT2* overexpression line (*35s:NIT2*, previously described by Normanly et al., 1997) did not have elevated IAA levels when grown under our standard conditions, however, it did have higher IAA levels compared to its genetic background (No-0) when grown under saline conditions (Figure 6A). While this could be because stress-inducible PTMs are required for activity, as suggested above, another reason could be altered IAA degradation which was observed in the *35s:NIT2* line. Specifically, *35s:NIT2* plants have increased IAA-Asp levels compared to the wildtype in untreated control conditions, but not in the presence of 100 mM NaCl (Figure 6B). The irreversible conjugation of Aspartic acid (Asp) to IAA tags it for oxidation and subsequent catabolism, and IAA-Asp conjugates are known to be involved in detoxification of excess IAA (Bajguz and Piotrowska, 2009). Thus, increased IAA detoxification may be needed in the *35s:NIT2* plants during growth in control conditions, but the increased IAA levels in saline environments may be beneficial and thus extra conjugation is no longer necessary. Unfortunately, we were unable to detect IAA-Asp in the Col-0 data (Figure 5). IAA-Asp is an extremely labile metabolite which is often difficult to measure (Novak et al., 2014).

As the *NIT2* overexpression line had increased IAA levels under saline conditions, we used a phenotypic approach to assess whether this was functionally important. It was somewhat perplexing that we did not observe altered salt tolerance in the two independent *nit2* T-DNA mutant lines (*nit2-1* and *nit2-2*) which have reduced *NIT2* expression (Supplementary Figures 3, 4), nor in the *nit2-*RNAi mutant line which has knocked down expression of all three *NIT-1* family genes (Lehmann et al., 2017) (Supplementary Figure 3, 4). While this may be due to compensation from alternative TD IAA biosynthetic pathways, it does suggest that NIT2 is not necessary for salt tolerance in wildtype Arabidopsis. Importantly, however, we showed that the *35s:NIT2* line, which has elevated IAA levels under saline conditions (Figure 6), also has improved survival and growth under saline conditions (Figures 7A-C). The fact that overexpression of *NIT2* did not improve plant growth under osmotic stress conditions further corroborates that *NIT2* is a salt-specific gene that does not compromise the plant response to osmotic stress (Figure 7B). Such a gene is an ideal candidate for improving plant growth under saline conditions and thereby improving plant salt tolerance.

Overexpression of *NIT2* and elevated IAA levels could sustain plant growth under saline conditions *via* auxin-mediated acid growth [reviewed by Arsuffi and Braybrook (2018)]. To explain this briefly; IAA induced signaling results in the accumulation of several SAUR proteins (Hagen and Guilfoyle, 2002), which in turn activate the plasma membrane (PM) H^+^-ATPase pump. This increased proton efflux results in apoplast acidification which activates enzymes involved in cell wall loosening and provides proton motive force to increase solute and water uptake which drives cell expansion and growth (Spartz et al., 2014). Not only would the enhanced cell expansion have a diluting effect on Na^+^ ions accumulated in the cytoplasm, but increased PM H^+^-ATPase activity would improve ion homeostasis and plant salt tolerance. For example, increased PM H^+^-ATPase activity would prevent membrane depolarization under saline conditions which, in turn, would prevent loss of crucial K^+^ and provide added driving force for high affinity K^+^ uptake, *via* KT/HAK/KUP transporters, and active Na^+^ extrusion, *via* Na^+^ antiporters such as SOS1 (Falhof et al., 2016; de Souza Miranda et al., 2017). Notably, we showed that under saline conditions the *35s:NIT2* line is better able to retain K^+^ in the root and prevent Na^+^ from accumulating in the shoot, thus maintaining a better Na:K ratio (Figure 7D and Supplementary Figure 6), supporting that the *35s:NIT2* line could have altered PM H^+^-ATPase activity. Several studies have already reported that constitutive activation of the PM H^+^-ATPase confers increased salt tolerance (Gévaudant et al., 2007; Janicka-Russak et al., 2013; Wang et al., 2013; Bose et al., 2015). Further work in this area should provide valuable mechanistic insights into how IAA modulates growth in saline conditions and improves salt tolerance.

To summarize, our work differentiates osmotic stress genes from salt-specific genes in Arabidopsis. A subset of salt-specific genes are auxin-responsive suggesting a function for auxin during plant growth in physiologically relevant saline conditions. We report elevated IAA levels in plants grown under saline conditions. Additionally, we identified an auxin biosynthetic gene, *NIT2*, which is specifically induced under saline conditions; and, when overexpressed, resulted in elevated IAA levels and improved growth specifically under saline conditions. Moreover, we provided evidence that NIT2 improves ionic stress tolerance and thus is a salt-specific gene. Salt-specific phenotypic improvement is a desirable trait for engineering plants with improved salt tolerance. Future work to understand how auxin improves plant growth under saline conditions and salt tolerance will allow us to apply these findings to crops.

## Data Availability Statement

The original contributions presented in the study are publicly available. These data can be found here: https://www.ncbi.nlm.nih.gov/geo/query/acc.cgi?acc=GSE193762.

## Author Contributions

LC performed the majority of the experimental work. CC and SM analyzed the data. PF performed the ion accumulation experiments. AP and ON performed the auxin metabolomics. CG and LD conceived the project. LC, RI, and LD drafted the manuscript. All authors contributed to editing the manuscript.

## Conflict of Interest

The authors declare that the research was conducted in the absence of any commercial or financial relationships that could be construed as a potential conflict of interest.

## Publisher’s Note

All claims expressed in this article are solely those of the authors and do not necessarily represent those of their affiliated organizations, or those of the publisher, the editors and the reviewers. Any product that may be evaluated in this article, or claim that may be made by its manufacturer, is not guaranteed or endorsed by the publisher.

## Footnotes

1
https://www.fao.org/3/i9553en/i9553en.pdf

## References

[B1] Abogadallah G. M. (2010). Sensitivity of *Trifolium alexandrinum* L. to salt stress is related to the lack of long-term stress-induced gene expression. *Plant Sci.* 178 491–500. 10.1016/j.plantsci.2010.03.008

[B2] Ahmad M. S. A. Javed F. Ashraf M. (2007). Iso-osmotic effect of NaCl and PEG on growth, cations and free proline accumulation in callus tissue of two indica rice (*Oryza sativa* L.) genotypes. *Plant Growth Regul.* 53 53–63. 10.1007/s10725-007-9204-0

[B3] Alarcón M. V. Salguero J. Lloret P. G. (2019). Auxin modulated initiation of lateral roots is linked to pericycle cell length in Maize. *Front. Plant Sci.* 10:11. 10.3389/fpls.2019.00011 30733725 PMC6354204

[B4] Alexa A. Rahnenführer J. Lengauer T. (2006). Improved scoring of functional groups from gene expression data by decorrelating GO graph structure. *Bioinformatics* 22 1600–1607. 10.1093/bioinformatics/btl140 16606683

[B5] Arsuffi G. Braybrook S. A. (2018). Acid growth: an ongoing trip. *J. Exp. Bot.* 69 137–146. 10.1093/jxb/erx390 29211894

[B6] Assaha D. V. M. Ueda A. Saneoka H. Al-Yahyai R. Yaish M. W. (2017). The role of Na+ and K+ transporters in salt stress adaptation in glycophytes. *Front. Physiol.* 8:509. 10.3389/fphys.2017.00509 28769821 PMC5513949

[B7] Bajguz A. Piotrowska A. (2009). Conjugates of auxin and cytokinin. *Phytochemistry* 70 957–969. 10.1016/j.phytochem.2009.05.006 19524990

[B8] Bartel B. Fink G. R. (1995). ILR1, an amidohydrolase that releases active indole-3-acetic acid from conjugates. *Science* 268 1745–1748. 10.1126/science.7792599 7792599

[B9] Bartling D. Seedorf M. Mithofer A. Weiler E. W. (1992). Cloning and expression of an *Arabidopsis nitrilase* which can convert indole-3-acetonitrile to the plant hormone, indole-3acetic acid. *Eur. J. Biochem.* 205 417–424. 10.1152/ajplung.1992.263.1.1-a 1555601

[B10] Bassil E. Zhang S. Gong H. Tajima H. Blumwald E. (2019). Cation specificity of vacuolar NHX-type cation/H + Antiporters 1[OPEN]. *Plant Physiol.* 179 616–629. 10.1104/pp.18.01103 30498025 PMC6426403

[B11] Benjamini Y. Hochberg Y. (1995). Controlling the false discovery rate - A practical and powerful approach to multiple testing. *J. R. Stat. Soc.* 1 289–300. 10.2307/2346101

[B12] Berens M. L. Berry H. M. Mine A. Argueso C. T. Tsuda K. (2017). Evolution of hormone signaling networks in plant defense. *Annu. Rev. Phytopathol.* 55 401–425. 10.1146/annurev-phyto-080516-035544 28645231

[B13] Bhatt D. Nath M. Sharma M. Bhatt M. D. Bisht D. S. Butani N. V. (2020). “Role of growth regulators and phytohormones in overcoming environmental stress,” in *Protective Chemical Agents in the Amelioration of Plant Abiotic Stress*, eds Roychoudhury A. Tripathi D. K. (Hoboken, NJ: John Wiley & Sons Ltd). 10.1002/9781119552154.ch11

[B14] Bose J. Rodrigo-Moreno A. Lai D. Xie Y. Shen W. Shabala S. (2015). Rapid regulation of the plasma membrane H+-ATPase activity is essential to salinity tolerance in two halophyte species, Atriplex lentiformis and *Chenopodium quinoa*. *Ann. Bot.* 115 481–494. 10.1093/aob/mcu219 25471095 PMC4332608

[B15] Calderon-Villalobos L. I. Tan X. Zheng N. Estelle M. (2010). Auxin perception — structural insights. *Cold Spring Harb. Perspect. Biol.* 2:a005546. 10.1101/cshperspect.a005546 20504967 PMC2890193

[B16] Cao X. Yang H. Shang C. Ma S. Liu L. Cheng J. (2019). The roles of auxin biosynthesis YUCCA gene family in plants. *Int. J. Mol. Sci.* 20:6343. 10.3390/ijms20246343 31888214 PMC6941117

[B17] Carillo P. Annunziata M. G. Pontecorvo G. Fuggi A. Woodrow P. (2011). “Salinity stress and salt tolerance,” in *Abiotic Stress in Plants- Mechanisms and Adaptations*, eds Shanker A. Venkateswaralu B. (Rijeka: In Tech).

[B18] Choi W. G. Toyota M. Kim S. H. Hilleary R. Gilroy S. (2014). Salt stress-induced Ca2+ waves are associated with rapid, long-distance root-to-shoot signaling in plants. *Proc. Natl. Acad. Sci. U.S.A.* 111 6497–6502. 10.1073/pnas.1319955111 24706854 PMC4035928

[B19] Cutler S. R. Somerville C. R. (2005). Imaging plant cell death: GFP-Nit1 aggregation marks an early step of wound and herbicide induced cell death. *BMC Plant Biol.* 5:4. 10.1186/1471-2229-5-4 15796778 PMC1087855

[B20] de Souza Miranda R. Mesquita R. O. Costa J. H. Alvarez-Pizarro J. C. Prisco J. T. Gomes-Filho E. (2017). Integrative control between proton pumps and SOS1 antiporters in roots is crucial for maintaining low Na+ accumulation and salt tolerance in ammonium-supplied *Sorghum bicolor*. *Plant Cell Physiol.* 58 522–536. 10.1093/pcp/pcw231 28158828

[B21] Dixon D. P. Skipsey M. Grundy N. M. Edwards R. (2005). Stress-induced protein S-glutathionylation in arabidopsis. *Plant Physiol.* 138 2233–2244. 10.1104/pp.104.058917 16055689 PMC1183410

[B22] Donaldson L. Ludidi N. Knight M. R. Gehring C. Denby K. (2004). Salt and osmotic stress cause rapid increases in *Arabidopsis thaliana* cGMP levels. *FEBS Lett.* 569 317–320. 10.1016/j.febslet.2004.06.016 15225654

[B23] Duan L. Dietrich D. Ng H. Yeen M. Bhalerao R. Bennett M. J. (2013). Endodermal ABA signaling promotes lateral root quiescence during salt stress in Arabidopsis seedlings. *Plant Cell* 25 324–341. 10.1105/tpc.112.107227 23341337 PMC3584545

[B24] Falhof J. Pedersen J. T. Fuglsang A. T. Palmgren M. (2016). Plasma Membrane H + -ATPase regulation in the center of plant physiology. *Mol. Plant* 9 323–337. 10.1016/j.molp.2015.11.002 26584714

[B25] FAO, IFAD, UNICEF, WFP, and WHO (2018). *The State of Food Security and Nutrition in the World 2018. Building Climate Resilience for Food Security and Nutrition*. Rome: FAO.

[B26] Feng W. Lindner H. Robbins N. E. Dinneny J. R. (2016). Growing out of stress: the role of cell- and organ-scale growth control in plant water-stress responses. *Plant Cell* 28 1769–1782. 10.1105/tpc.16.00182 27503468 PMC5006702

[B27] Fu Y. Yang Y. Chen S. Ning N. Hu H. (2019). Arabidopsis IAR4 modulates primary root growth under salt stress through ROS-mediated modulation of auxin distribution. *Front. Plant Sci.* 10:522. 10.3389/fpls.2019.00522 31105724 PMC6494962

[B28] Galvan-Ampudia C. S. Julkowska M. M. Darwish E. Gandullo J. Korver R. A. Brunoud G. (2013). Halotropism is a response of plant roots to avoid a saline environment. *Curr. Biol.* 23 2044–2050. 10.1016/j.cub.2013.08.042 24094855

[B29] Gévaudant F. Duby G. Von Stedingk E. Zhao R. Morsomme P. Boutry M. (2007). Expression of a constitutively activated plasma membrane H +-ATPase alters plant development and increases salt tolerance. *Plant Physiol.* 144 1763–1776. 10.1104/pp.107.103762 17600134 PMC1949876

[B30] Goyal E. Amit S. K. Singh R. S. Mahato A. K. Chand S. Kanika K. (2016). Transcriptome profiling of the salt-stress response in *Triticum aestivum* cv. Kharchia Local. *Sci. Rep.* 6:27752. 10.1038/srep27752 27293111 PMC4904219

[B31] Gray W. M. (2004). Hormonal regulation of plant growth and development. *PLoS Biol.* 2:e311. 10.1371/journal.pbio.0020311 15367944 PMC516799

[B32] Grsic S. Sauerteig S. Neuhaus K. Albrecht M. Rossiter J. Ludwig-Muller J. (1998). Physiological analysis of transgenic *Arabidopsis thaliana* plants expressing one nitrilase isoform in sense or antisense direction. *J. Plant Physiol.* 153 446–456. 10.1016/S0176-1617(98)80173-9

[B33] Hagen G. Guilfoyle T. (2002). Auxin-responsive gene expression?: genes, promoters and regulatory factors. *Plant Mol. Biol.* 49 373–385. 12036261

[B34] Haugh G. W. Sommerville C. (1986). Sulfonylurea-resistant mutants of *Arabidopsis thaliana*. *Mol. Gen. Genet.* 204 430–434.

[B35] Hong S. M. Bahn S. C. Lyu A. Jung H. S. Ahn J. H. (2010). Identification and testing of superior reference genes for a starting pool of transcript normalization in Arabidopsis. *Plant Cell Physiol.* 51 1694–1706. 10.1093/pcp/pcq128 20798276

[B36] Howe E. Holton K. Nair S. Schlauch D. Sinha R. Quackenbush J. (2010). “MeV: multiexperiment viewer,” in *Biomedical Informatics for Cancer Research*, Vol. 15 eds Ochs M. Casagrande J. Davuluri R. (Boston, MA: Springer), 267–277. 10.1007/978-1-4419-5714-6

[B37] Iglesias M. J. Terrile M. C. Windels D. Lombardo M. C. Bartoli C. G. Vazquez F. (2014). MiR393 regulation of auxin signaling and redox-related components during acclimation to salinity in Arabidopsis. *PLoS One* 9:e107678. 10.1371/journal.pone.0107678 25222737 PMC4164656

[B38] Iqbal M. Ashraf M. (2007). Seed treatment with auxins modulates growth and ion partitioning in salt - stressed wheat plants seed treatment with auxins modulates growth and ion partitioning in salt-stressed wheat plants. *J. Integr. Plant Biol.* 49 1003–1015. 10.1111/j.1672-9072.2007.00488.x

[B39] Isayenkov S. V. Maathuis F. J. M. (2019). Plant salinity stress: many unanswered questions remain. *Front. Plant Sci.* 10:80. 10.3389/fpls.2019.00080 30828339 PMC6384275

[B40] Ivanchenko M. G. Napsucialy-Mendivil S. Dubrovsky J. G. (2010). Auxin-induced inhibition of lateral root initiation contributes to root system shaping in *Arabidopsis thaliana*. *Plant J.* 64 740–752. 10.1111/j.1365-313X.2010.04365.x 21105922

[B41] Ivushkin K. Bartholomeus H. Bregt A. K. Pulatov A. Kempen B. de Sousa L. (2019). Global mapping of soil salinity change. *Remote Sens. Environ.* 231:111260. 10.1016/j.rse.2019.111260

[B42] Janicka-Russak M. Kabała K. Wdowikowska A. Kłobus G. (2013). Modification of plasma membrane proton pumps in cucumber roots as an adaptation mechanism to salt stress. *J. Plant Physiol.* 170 915–922. 10.1016/j.jplph.2013.02.002 23499455

[B43] Jenrich R. Trompetter I. Bak S. Olsen C. E. Moller B. L. Piotrowski M. (2007). Evolution of heteromeric nitrilase complexes in Poaceae with new functions in nitrile metabolism. *Proc. Natl. Acad. Sci. U.S.A.* 104 18848–18853. 10.1073/pnas.0709315104 18003897 PMC2141865

[B44] Ji H. Pardo J. M. Batelli G. Van Oosten M. J. Bressan R. A. Li X. (2013). The salt overly sensitive (SOS) pathway: established and emerging roles. *Mol. Plant* 6 275–286. 10.1093/mp/sst017 23355543

[B45] Jiang Y. Deyholos M. K. (2006). Comprehensive transcriptional profiling of NaCl-stressed Arabidopsis roots reveals novel classes of responsive genes. *BMC Plant Biol.* 6:25. 10.1186/1471-2229-6-25 17038189 PMC1621065

[B46] Julkowska M. M. Testerink C. (2015). Tuning plant signaling and growth to survive salt. *Trends Plant Sci.* 20 586–594. 10.1016/j.tplants.2015.06.008 26205171

[B47] Julkowska M. M. Hoefsloot H. C. J. Mol S. Feron R. de Boer G. J. Haring M. A. (2014). Capturing Arabidopsis root architecture dynamics with ROOT - FIT reveals diversity in responses to salinity. *Plant Physiol.* 166 1387–1402. 10.1104/pp.114.248963 25271266 PMC4226346

[B48] Jung J.-H. Park C.-M. (2011). Auxin modulation of salt stress signaling in Arabidopsis seed germination. *Plant Signal. Behav.* 6 1198–1200. 10.4161/psb.6.8.15792 21757997 PMC3260721

[B49] Kasahara H. (2016). Current aspects of auxin biosynthesis in plants. *Biosci. Biotechnol. Biochem.* 80 34–42. 10.1080/09168451.2015.1086259 26364770

[B50] Klepek Y. S. Volke M. Konrad K. R. Wippel K. Hoth S. Hedrich R. (2010). *Arabidopsis thaliana* POLYOL/MONOSACCHARIDE TRANSPORTERS 1 and 2: fructose and xylitol/H+ symporters in pollen and young xylem cells. *J. Exp. Bot.* 61 537–550. 10.1093/jxb/erp322 19969532 PMC2803217

[B51] Koevoets I. T. Venema J. H. Elzenga J. T. M. Testerink C. (2016). Roots withstanding their environment: exploiting root system architecture responses to abiotic stress to improve crop tolerance. *Front. Plant Sci.* 7:1335. 10.3389/fpls.2016.01335 27630659 PMC5005332

[B52] Korver R. A. Koevoets I. T. Testerink C. (2018). Out of Shape during stress: a key role for auxin. *Trends Plant Sci.* 23 783–793. 10.1016/j.tplants.2018.05.011 29914722 PMC6121082

[B53] Ku Y.-S. Sintaha M. Cheung M.-Y. Lam H.-M. (2018). Plant hormone signaling crosstalks between biotic and abiotic stress responses. *Int. J. Mol. Sci.* 19:3206. 10.3390/ijms19103206 30336563 PMC6214094

[B54] Lehmann T. Janowitz T. Sánchez-Parra B. Alonso M.-M. P. Trompetter I. Piotrowski M. (2017). Arabidopsis NITRILASE 1 Contributes to the regulation of root growth and development through modulation of auxin biosynthesis in seedlings. *Front. Plant Sci.* 8:36. 10.3389/fpls.2017.00036 28174581 PMC5258727

[B55] Li X. Li M. Zhou B. Yang Y. Wei Q. Zhang J. (2019). Transcriptome analysis provides insights into the stress response crosstalk in apple (*Malus × domestica*) subjected to drought, cold and high salinity. *Sci. Rep.* 9:9071. 10.1038/s41598-019-45266-0 31227734 PMC6588687

[B56] Liu W. Li R.-J. Han T.-T. Cai W. Fu Z.-W. Lu Y.-T. (2015). Salt Stress reduces root meristem size by nitric oxide-mediated modulation of auxin accumulation and signaling in Arabidopsis. *Plant Physiol.* 168 343–356. 10.1104/pp.15.00030 25818700 PMC4424022

[B57] Liu Y. Ji X. Zheng L. Nie X. Wang Y. (2013). Microarray analysis of transcriptional responses to abscisic acid and salt stress in *Arabidopsis thaliana*. *Int. J. Mol. Sci.* 14 9979–9998. 10.3390/ijms14059979 23665901 PMC3676824

[B58] Ljung K. (2013). Auxin metabolism and homeostasis during plant development. *Development* 140 943–950. 10.1242/dev.086363 23404103

[B59] Mann H. B. Whitney D. R. (1947). On a test of whether one of two random variables is stochastically larger than the other. *Ann. Math. Stat.* 18 50–60. 10.1214/aoms/1177730491

[B60] Morton M. J. L. Awlia M. Al-Tamimi N. Saade S. Pailles Y. Negrão S. (2019). Salt stress under the scalpel – dissecting the genetics of salt tolerance. *Plant J.* 97 148–163. 10.1111/tpj.14189 30548719 PMC6850516

[B61] Munns R. (2002). Comparative physiology of salt and water stress. *Plant Cell Environ.* 25 239–250. 10.1046/j.0016-8025.2001.00808.x 11841667

[B62] Munns R. Tester M. (2008). Mechanisms of salinity tolerance. *Annu. Rev. Plant Biol.* 59 651–681. 10.1146/annurev.arplant.59.032607.092911 18444910

[B63] Munns R. James R. A. Xu B. Athman A. Conn S. J. Jordans C. (2012). Wheat grain yield on saline soils is improved by an ancestral Na^+^ transporter gene. *Nat. Biotechnol.* 30 360–364. 10.1038/nbt.2120 22407351

[B64] Naser V. Shani E. (2016). Auxin response under osmotic stress. *Plant Mol. Biol.* 91 661–672. 10.1007/s11103-016-0476-5 27052306

[B65] Negrão S. Schmöckel S. M. Tester M. (2017). Evaluating physiological responses of plants to salinity stress. *Ann. Bot.* 119 1–11. 10.1093/aob/mcw191 27707746 PMC5218372

[B66] Normanly J. Grisafi P. Fink G. R. Barteld B. (1997). Arabidopsis mutants resistant to the auxin effects of indole-3-acetonitrile are defective in the nitrilase encoded by the NITI Gene Col-0. *Plant Cell* 9 1781–1790. 10.1105/tpc.9.10.1781 9368415 PMC157021

[B67] Novák O. Hényková E. Sairanen I. Kowalczyk M. Pospíšil T. Ljung K. (2012). Tissue-specific profiling of the *Arabidopsis thaliana* auxin metabolome. *Plant J.* 72 523–536. 10.1111/j.1365-313X.2012.05085.x 22725617

[B68] Novak S. D. Luna L. J. Gamage R. N. (2014). Role of auxin in orchid development. *Plant Signal. Behav.* 9:e972277. 10.4161/psb.32169 25482818 PMC4622584

[B69] Overvoorde P. Fukaki H. Beeckman T. (2010). Auxin control of root development. *Cold Spring Harb. Perspect. Biol.* 2:a001537. 10.1101/cshperspect.a001537 20516130 PMC2869515

[B70] Pavlović I. Pěnčík A. Novák O. Vujčić V. Radić Brkanac S. Lepeduš H. (2018). Short-term salt stress in *Brassica rapa* seedlings causes alterations in auxin metabolism. *Plant Physiol. Biochem.* 125 74–84. 10.1016/j.plaphy.2018.01.026 29427890

[B71] Piotrowski M. Schönfelder S. Weiler E. W. (2001). The *Arabidopsis thaliana* Isogene NIT4 and Its orthologs in tobacco encode ??-Cyano-L-alanine Hydratase/Nitrilase. *J. Biol. Chem.* 276 2616–2621. 10.1074/jbc.M007890200 11060302

[B72] Prakash L. Prathapasenan G. (1990). NaCl-and gibberellic acid-induced changes in the content of auxin and the activities of cellulase and pectin lyase during leaf growth in rice (*Oryza sativa*). *Ann. Bot.* 65 251–257.

[B73] Prerostova S. Dobrev P. I. Gaudinova A. Hosek P. Soudek P. Knirsch V. (2017). Hormonal dynamics during salt stress responses of salt-sensitive *Arabidopsis thaliana* and salt-tolerant *Thellungiella salsuginea*. *Plant Sci.* 264 188–198. 10.1016/j.plantsci.2017.07.020 28969799

[B74] Ryu H. Cho Y.-G. (2015). Plant hormones in salt stress tolerance. *J. Plant Biol.* 58 147–155. 10.1007/s12374-015-0103-z

[B75] Schmidt R. C. Müller A. Hain R. Bartling D. Weiler E. W. (1996). Transgenic tobacco plants expressing the *Arabidopsis thaliana* nitrilase II enzyme. *Plant J.* 9 683–691. 10.1046/j.1365-313X.1996.9050683.x 8653117

[B76] Shabala S. Cuin T. (2007). Potassium transport and plant salt tolerance. *Physiol. Plant.* 133 651–669. 10.1111/j.1399-3054.2007.01008.x 18724408

[B77] Shavrukov Y. (2013). Salt stress or salt shock: which genes are we studying? *J. Exp. Bot.* 64 119–127. 10.1093/jxb/ers316 23186621

[B78] Spartz A. K. Ren H. Park M. Y. Grandt K. N. Lee H. Murphy A. S. (2014). SAUR inhibition of PP2C-D phosphatases activates plasma membrane H + -ATPases to promote cell expansion in Arabidopsis. *Plant Cell* 26 2129–2142. 10.1105/tpc.114.126037 24858935 PMC4079373

[B79] Sun F. Zhang W. Hu H. Li B. Wang Y. Zhao Y. (2008). Salt modulates gravity signaling pathway to regulate growth direction of primary roots in Arabidopsis. *Plant Physiol.* 146 178–188. 10.1104/pp.107.109413 18024552 PMC2230569

[B80] Tang M. Liu X. Deng H. Shen S. (2011). Over-expression of JcDREB, a putative AP2/EREBP domain-containing transcription factor gene in woody biodiesel plant *Jatropha curcas*, enhances salt and freezing tolerance in transgenic *Arabidopsis thaliana*. *Plant Sci.* 181 623–631. 10.1016/j.plantsci.2011.06.014 21958703

[B81] Tani E. Sarri E. Goufa M. Asimakopoulou G. Psychogiou M. Bingham E. (2018). Seedling growth and transcriptional responses to salt shock and stress in *Medicago sativa* L., *Medicago arborea* L., and Their Hybrid (Alborea). *Agronomy* 8:231. 10.3390/agronomy8100231

[B82] Tilbrook J. Roy S. (2014). “Salinity tolerance,” in *Plant Abiotic Stress*, 2nd Edn, eds Jenks M. A. Hasegawa P. M. (New York, NY: Wiley-Blackwell), 134–178.

[B83] van den Berg T. Korver R. A. Testerink C. ten Tusscher K. H. W. J. (2016). Modeling halotropism: a key role for root tip architecture and reflux loop remodeling in redistributing auxin. *Development* 143 3350–3362. 10.1242/dev.135111 27510970 PMC5047658

[B84] Van Zelm E. Zhang Y. Testerink C. (2020). Salt tolerance mechanisms of plants. *Annu. Rev. Plant Biol.* 71 403–433. 10.1146/annurev-arplant-050718-100005 32167791

[B85] Verma V. Ravindran P. Kumar P. P. (2016). Plant hormone-mediated regulation of stress responses. *BMC Plant Biol.* 16:86. 10.1186/s12870-016-0771-y 27079791 PMC4831116

[B86] Vorwerk S. Biernacki S. Hillebrand H. Janzik I. Müller A. Weiler E. W. (2001). Enzymatic characterization of the recombinant *Arabidopsis thaliana* nitrilase subfamily encoded by the NIT2/NIT1/NIT3-gene cluster. *Planta* 212 508–516. 10.1007/s004250000420 11525507

[B87] Wang F. Chen Z.-H. Shabala S. (2017). Hypoxia sensing in plants: on a quest for ion channels as putative oxygen sensors. *Plant Cell Physiol.* 58 1126–1142. 10.1093/pcp/pcx079 28838128

[B88] Wang M. Wang Y. Sun J. Ding M. Deng S. Hou P. (2013). Overexpression of PeHA1 enhances hydrogen peroxide signaling in salt-stressed Arabidopsis. *Plant Physiol. Biochem.* 71 37–48. 10.1016/j.plaphy.2013.06.020 23872741

[B89] Wang P. Shen L. Guo J. Jing W. Qu Y. Li W. (2019). Phosphatidic acid directly regulates PINOID-dependent phosphorylation and activation of the PIN-FORMED 2 auxin efflux transporter in response to salt stress. *Plant Cell* 31 250–271. 10.1105/tpc.18.00528 30464035 PMC6391703

[B90] Wang Y. Li K. Li X. (2009). Auxin redistribution modulates plastic development of root system architecture under salt stress in Arabidopsis thaliana. *J. Plant Physiol.* 166 1637–1645. 10.1016/j.jplph.2009.04.009 19457582

[B91] Wilson A. K. Pickett F. B. Turner J. C. Estelle M. (1990). A dominant mutation in Arabidopsis confers resistance to auxin, ethylene and abscisic acid. *Mol. Gen. Genet.* 222 377–383. 10.1007/BF00633843 2148800

[B92] Woodward J. D. Trompetter I. Sewell B. T. Piotrowski M. (2018). Substrate specificity of plant nitrilase complexes is affected by their helical twist. *Commun. Biol.* 1:186. 10.1038/s42003-018-0186-4 30417123 PMC6214922

[B93] Yang J. Duan G. Li C. Liu L. Han G. Zhang Y. (2019). The crosstalks between jasmonic acid and other plant hormone signaling highlight the involvement of jasmonic acid as a core component in plant response to biotic and abiotic stresses. *Front. Plant Sci.* 10:1349. 10.3389/fpls.2019.01349 31681397 PMC6813250

[B94] Yang Y. Guo Y. (2018). Elucidating the molecular mechanisms mediating plant salt-stress responses. *New Phytol.* 217 523–539. 10.1111/nph.14920 29205383

[B95] Zhao Y. (2018). Essential roles of local auxin biosynthesis in plant development and in adaptation to environmental changes. *Annu. Rev. Plant Biol.* 69 417–435. 10.1146/annurev-arplant-042817-040226 29489397

[B96] Zhao Y. Wang T. Zhang W. Li X. (2011). SOS3 mediates lateral root development under low salt stress through regulation of auxin redistribution and maxima in Arabidopsis. *New Phytol.* 189 1122–1134. 10.1111/j.1469-8137.2010.03545.x 21087263

[B97] Zolla G. Heimer Y. M. Barak S. (2010). Mild salinity stimulates a stress-induced morphogenic response in *Arabidopsis thaliana* roots. *J. Exp. Bot.* 61 211–224. 10.1093/jxb/erp290 19783843 PMC2791118

[B98] Zörb C. Geilfus C. M. Dietz K. J. (2019). Salinity and crop yield. *Plant Biol.* 21 31–38. 10.1111/plb.12884 30059606

