# Supplementary material for: Salt-Specific Gene Expression Reveals Elevated Auxin Levels in Arabidopsis thaliana Plants Grown Under Saline Conditions
Source: Front Plant Sci. 2022 Feb 10;13:804716. doi: 10.3389/fpls.2022.804716 (PMC8866861; doi:10.3389/fpls.2022.804716)
Supplement: Supplementary file 1 [file Data_Sheet_1.zip › Supplementary Figures.docx]

**Supplementary figures**

**A**

**B**


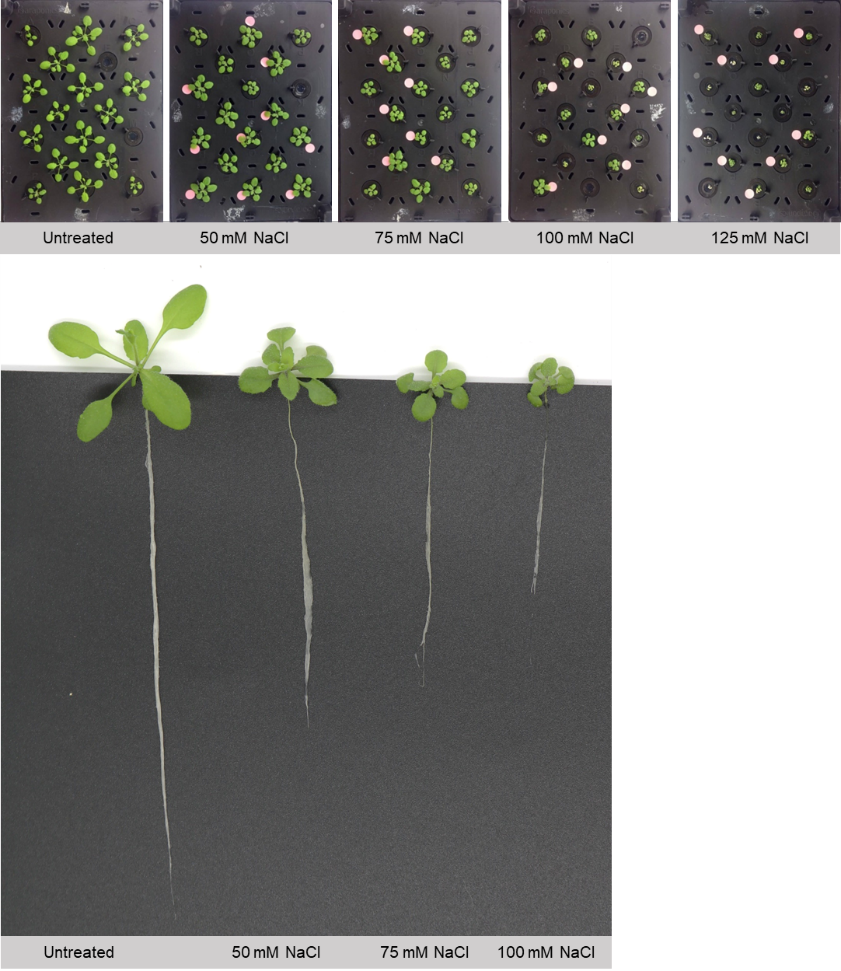


**Supplementary figure 1: Testing the impact of different NaCl treatment concentrations on plant growth and development to confirm relevant treatment concentrations for further experiments**. Col-0 was germinated and grown hydroponically in untreated control ATS media for two weeks. Seedlings were then transferred onto ATS media supplemented with 0, 50, 75, 100 or 125 mM NaCl and grown in the salt treatment until the plants reached vegetative reproductive stage. Plants grown in the 125 mM NaCl treatment did not reach vegetative reproductive stage **A:** Four-week-old Arabidopsis plants after two weeks of growth in different NaCl concentrations**. B:** Four-week-old Arabidopsis plants after two weeks of growth in different NaCl concentrations illustrating the impact on root and shoot growth.

**
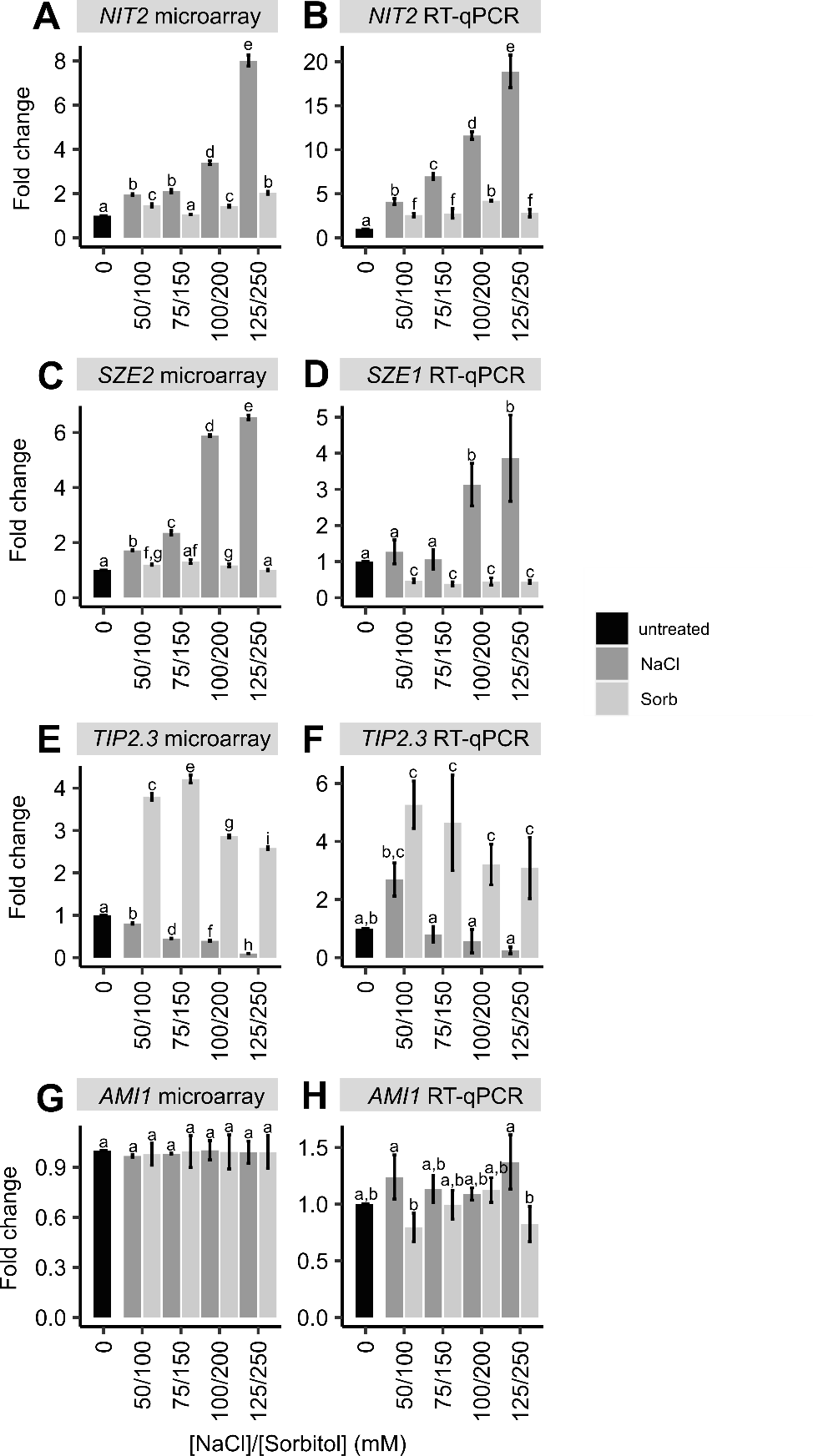
**

**Supplementary figure 2: RT-qPCR validation of the microarray data.** Arabidopsis Col-0 was germinated and grown for two weeks in petri dishes containing ATS media (0 mM, untreated control) and ATS supplemented with the indicated concentrations of NaCl and sorbitol (Sorb). Gene expression was determined by microarray analysis (**A**, **C**, **E** and **G**). Five genes, two with salt-specific expression, one with osmotic specific expression and one with no change in expression were chosen to validate the microarray data by RT-qPCR (**B**, **D**, **F** and **H**). The black bar represents the untreated control, dark grey bars represent NaCl treatments and light grey bars represent sorbitol treatments.Results are shown as the mean fold change relative to the untreated control (0 mM). The mean was calculated from three biological repeats. Error bars represent standard error. Different letters on the graphs indicate statistically significant differences (*p* ≤ 0.05) in mean fold change (nmol.g^-1^ DW) as determined by one way ANOVA followed by Fisher LSD post-hoc analysis.. The RNA used for the microarray and RT-qPCR experiments was extracted from plants grown in independent experiments. The RT-qPCR results were normalized to the *SAND* reference gene. ***NIT2*:** *Nitrilase 2,* ***SZE2*:** *SUPPRESSOR OF ZED1-D2,* ***TIP2.3:*** *TONOPLAST INTRINSIC PROTEIN 2.3,* ***AMI1*:** *Indole-3-acetamide 1*.

*
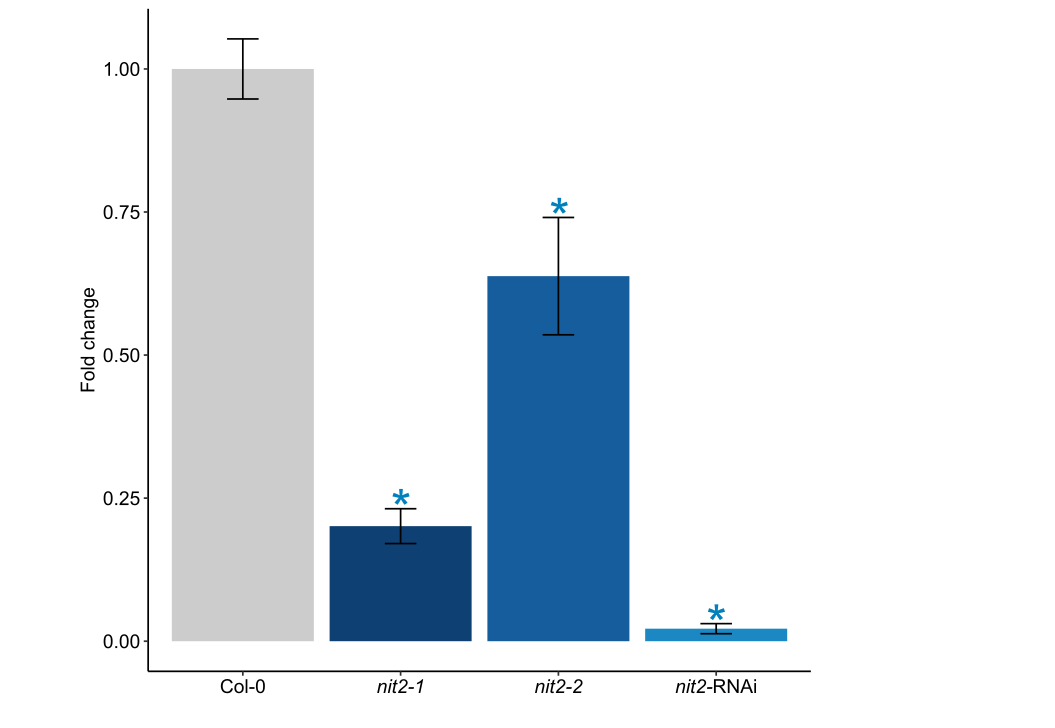
*

**Supplementary figure 3: Confirmation of *NIT2* expression in mutant plant lines.** The *nit2-1* and *nit2-2* lines are T-DNA insertion mutants while the *nit2*-RNAi line has been previously described (Lehmann et al., 2017). RNA for cDNA synthesis and subsequent RT-qPCR was extracted from a single leaf from three individual plants. The RT-qPCR results were normalized to the *SAND* reference gene. The average fold change in *NIT2* expression (calculated from 3 biological repeats) is shown relative to the Col-0 wild type. The grey bar represents Col-0 and blue bars represent the *nit2* mutant lines. Error bars indicate standard error. A star indicates a statistically significant difference in fold change between Col-0 and *nit2* mutant line*.* Significance was determined by independent t-tests, with a *p*-value ≤ 0.05 considered significant.

**
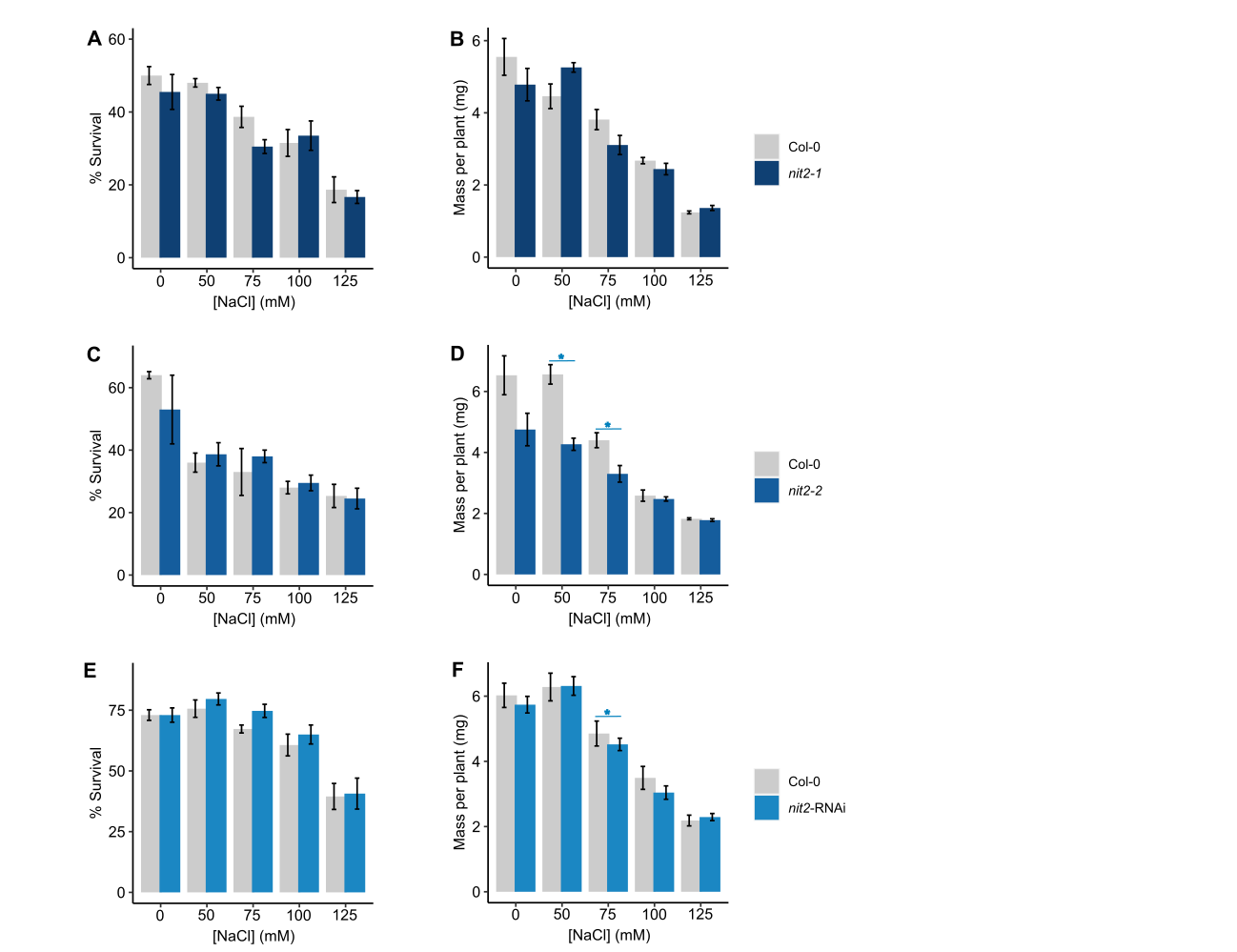
**

**Supplementary figure 4: Functional characterisation of the Arabidopsis *nit2* mutant lines grown on NaCl.**Col-0 (wildtype) and *nit2-1*, *nit2-2* (T-DNA insertion lines) or *nit2-*RNAi (Lehmann et al., 2017) plants were germinated and grown for two weeks in petri dishes containing ATS media (0 mM, untreated control) and ATS supplemented with the indicated concentrations of NaCl, and survival and plant growth (mass per plant) determined. **A:** The average % survival of Col-0 and *nit2-1*. **B:** The average mass per plant of Col-0 and *nit2-1.* **C:** The average % survival of Col-0 and *nit2-2*. **D:** The average mass per plant of Col-0 and *nit2-2.* **E:** The average % survival of Col-0 and *nit2-*RNAi. **F:** The average mass per plant of Col-0 and *nit2-*RNAi. The grey bars represent Col-0 and blue bars represent the *nit2* mutant lines. The results for each plant line are an average of three independent experiments. Error bars represent standard error. A star indicates a statistically significant difference in mean survival/mass between the Col-0 and the mutant in that treatment. Significance was determined by independent t-tests with a corrected *p*-value ≤ 0.05 considered significant.. The Bonferroni method was used to correct *p*-values for multiple testing.


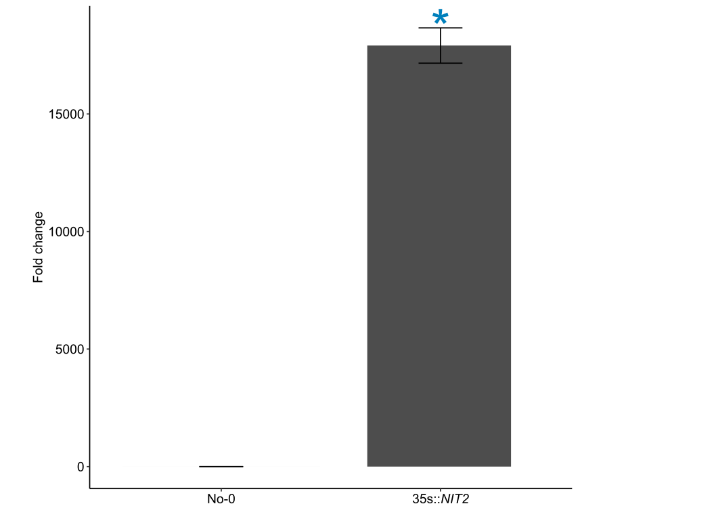


**Supplementary figure 5: Confirmation of *NIT2* overexpression in *35s::NIT2* plants.** The *35s::NIT2* line and its wildtype background (No-0) have previously been described by Normanly et al., 1997. RNA for cDNA synthesis and subsequent RT-qPCR was extracted from a single leaf from individual plants grown in untreated control conditions. The average fold change in *NIT2* expression is shown relative to the No-0 wild type. The average was calculated from two biological repeats, each of which was analysed in three technical repeats. Error bars indicate standard error. A star indicates a statistically significant difference in fold change between No-0 and 35s::*NIT2.* Significance was determined by independent t-tests, with a *p*-value ≤ 0.05 considered significant. The RT-qPCR results were normalized to the *SAND* reference gene.


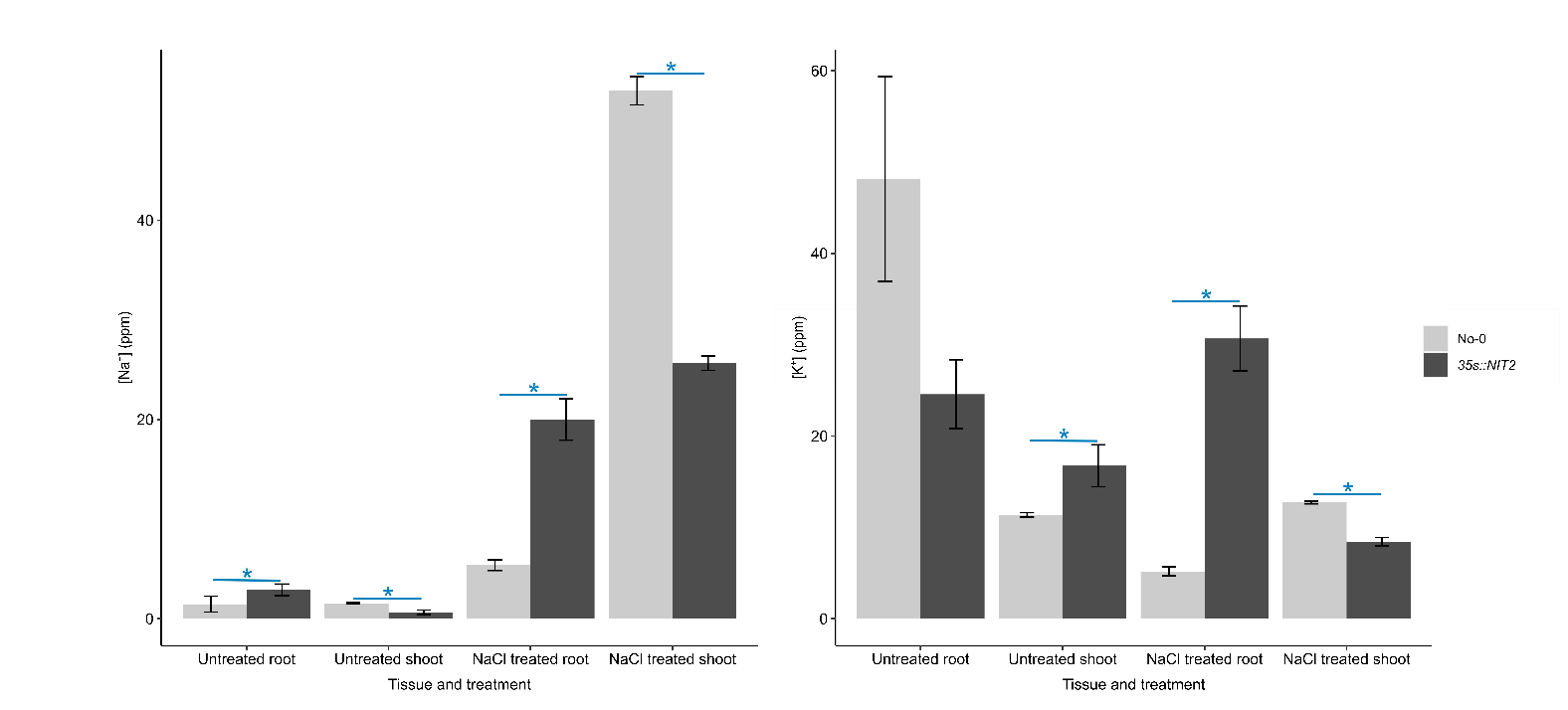


**B**

**A**

**Supplementary figure 6: The concentrations of Na^+^  and K^+^ ions in** **untreated and NaCl treated root and shoot tissue from No-0 and 35s::*NIT2* plants.** No-0 and 35s::*NIT2* plants were grown hydroponically for three weeks on ¼ strength ATS media then transferred onto ¼ strength ATS media supplemented with 0 mM or 75 mM NaCl for one week further. The Na^+^ (**A**) and K^+^ (**B**) content in shoot and root tissue was determined via ICP-OES analysis. The results are the mean of six biological repeats. The light grey bars represent No-0 and dark grey bars represent *35s::NIT2*. Error bars represent standard error. A star indicates a statistically significant difference in mean between No-0 and 35s::*NIT2* in that treatment/tissue. Significance was determined by independent t-tests with a corrected *p*-value ≤ 0.05 considered significant. The Bonferroni method was used to correct *p*-values for multiple testing.
